# Minimal aromatic aldehyde reduction (MARE) yeast platform for engineering vanillin production

**DOI:** 10.1186/s13068-023-02454-5

**Published:** 2024-01-06

**Authors:** Qiwen Mo, Jifeng Yuan

**Affiliations:** State Key Laboratory of Cellular Stress Biology, School of Life Sciences, Faculty of Medicine and Life Sciences, Xiamen University, Fujian, 361102 China

**Keywords:** Vanillin, Aldehyde stability, Pathway engineering, Synthetic biology

## Abstract

**Background:**

Vanillin represents one of the most widely used flavoring agents in the world. However, microbial synthesis of vanillin is hindered by the host native metabolism that could rapidly degrade vanillin to the byproducts.

**Results:**

Here, we report that the industrial workhorse *Saccharomyces cerevisiae* was engineered by systematic deletion of oxidoreductases to improve the vanillin accumulation. Subsequently, we harnessed the minimal aromatic aldehyde reduction (MARE) yeast platform for de novo synthesis of vanillin from glucose. We investigated multiple coenzyme-A free pathways to improve vanillin production in yeast. The vanillin productivity in yeast was enhanced by multidimensional engineering to optimize the supply of cofactors (NADPH and *S*-adenosylmethionine) together with metabolic reconfiguration of yeast central metabolism. The final yeast strain with overall 24 genetic modifications produced 365.55 ± 7.42 mg l^−1^ vanillin in shake-flasks, which represents the best reported vanillin titer from glucose in yeast.

**Conclusions:**

The success of vanillin overproduction in budding yeast showcases the great potential of synthetic biology for the creation of suitable biocatalysts to meet the requirement in industry. Our work lays a foundation for the future implementation of microbial production of aromatic aldehydes in budding yeast.

**Graphical Abstract:**

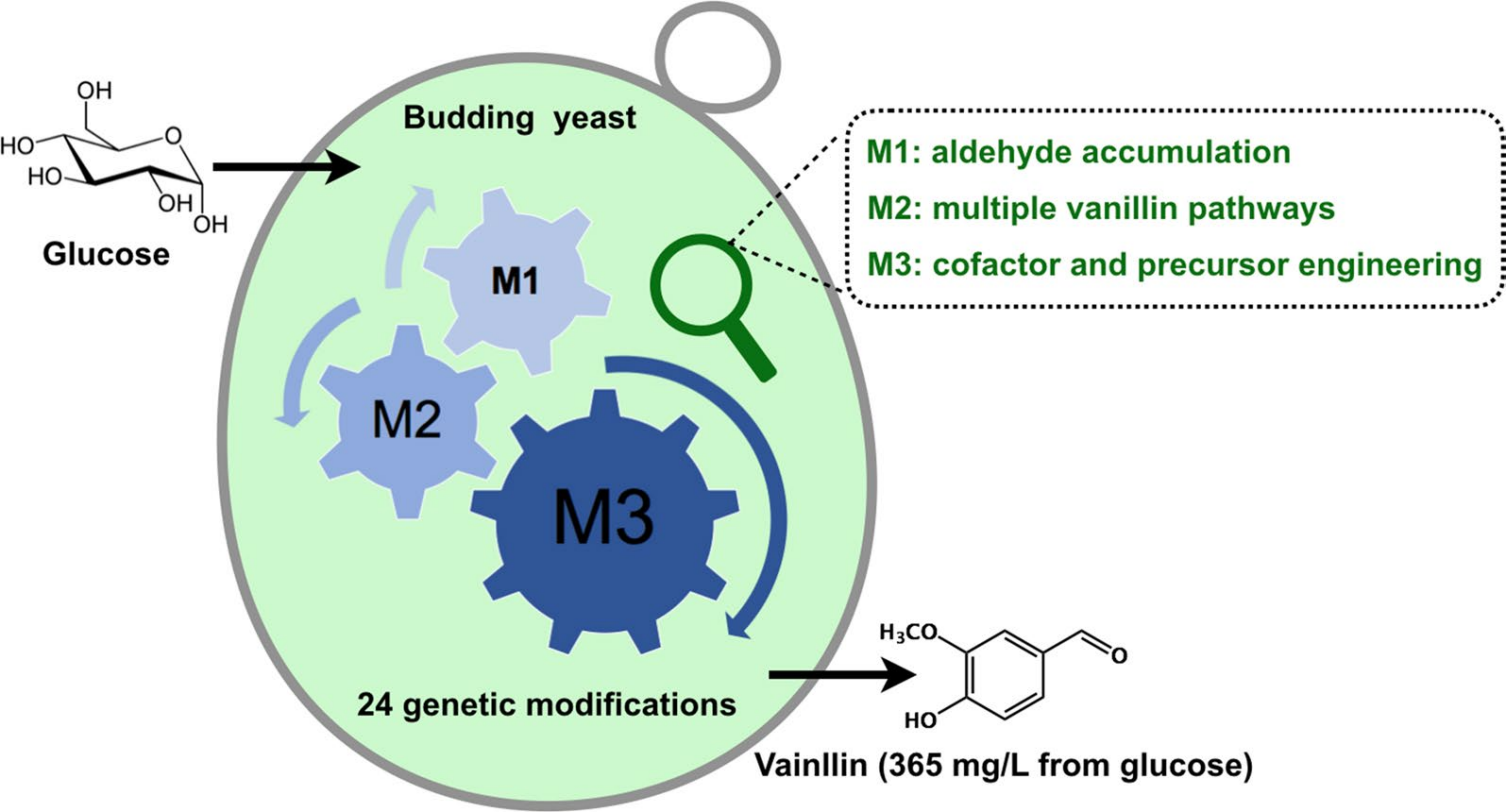

**Supplementary Information:**

The online version contains supplementary material available at 10.1186/s13068-023-02454-5.

## Background

Aromatic aldehydes are used widely as flavors and fragrances, and serve as intermediates for alkaloid synthesis [1, 2]. For instance, vanillin has an intense and tenacious creamy vanilla-like taste, which makes it one of the most widely used flavoring agents in the world [3]. As a plant secondary metabolite, vanillin can be extracted from the seedpods of orchids such as *Vanilla*. Because of the vanilla orchid's sluggish development and very low vanillin concentration in the mature vanilla pods, the plant-sourced vanillin comes with a relatively high cost (US$ 515 kg^−1^ in June 2018) [3, 4]. Although vanillin can be synthesized from fossil hydrocarbons with a cheap price (approximately US$ 12 kg^−1^) [3, 4], the chemically synthesized vanillin is not suitable for food and beverage industry. Hence, the price of “natural” vanillin is almost 250 times higher than the synthetic vanillin [5].

Microbial synthesized vanillin from natural substrates is classified as “natural” vanillin under European and US food legislation [6, 7]. For instance, ferulic acid and eugenol are commonly realized as substrates for the biocatalytic production of vanillin [8–13]. In comparison, de novo biosynthesis of vanillin from simple sugars such as glucose is a more attractive alternative because sugars are less expensive than ferulic acid and eugenol. The de novo synthetic pathway in the vanilla plant involves the phenylpropanoid pathway for converting glucose into ferulic acid, and a hydratase/lyase-type enzyme known as vanillin synthase (VpVAN) to catalyze the conversion of ferulic acid to vanillin glycoside [14]. Frost et al. harnessed the recombinant *Escherichia coli* for synthesis of vanillin from glucose via a two-step approach [15]: vanillate was first produced by fermentation, and it was then reduced to vanillin by aryl aldehyde dehydrogenase in vitro. Mimicking the natural vanillin pathway from plants was also established in *E. coli* and 19.3 mg l^−1^ vanillin was achieved from glucose [16]. By taking advantage of the native shikimate pathway of *E. coli*, Li et al*.* pioneered a new de novo synthetic pathway for vanillin production by directing the flux towards protocatechuate [9]. Through this pathway, genetically modified yeast strains, *Schizosaccharomyces pombe* and *Saccharomyces cerevisiae*, achieved the accumulations of 65 mg l^−1^ and 45 mg l^−1^ vanillin, respectively [17]. However, the main barrier to microbial synthesis of vanillin is the rapid conversion into its corresponding alcohol. The metabolic engineering strategy to minimize non-specific aldehyde reductase activities is therefore required.

With the development of synthetic biology and metabolic engineering, researchers began to focus on addressing the instability issue of vanillin caused by the host metabolism. Most microorganisms could not naturally accumulate aldehydes because of the endogenous alcohol dehydrogenases (ADHs), aldo–keto reductases (AKRs), and aldehyde reductases (ALDRs). For example, Kunjapur et al. employed *E. coli* with reduced aldehyde reduction as a platform for aromatic aldehyde biosynthesis [18]. After introducing the vanillin biosynthetic pathway, the engineered *E. coli* produced 119 mg l^−1^ vanillin from glucose [18]. Jin-Ho Lee et al*.* identified that a single gene knockout of *NCgl0324* in *Corynebacterium glutamicum* could substantially improve the production of protocatechualdehyde (PCAL) and vanillin [19]. High-level vanillin production not only requires a reduced aldehyde reduction, but also an optimal metabolic pathway. For instance, Kunjapur et al*.* [20] identified a key bottleneck in vanillin biosynthesis involving the methylation of protocatechuate to vanillate. By modifying the *metJ* gene, and overexpressing *metA*-*cysE* genes, the supply of *S*-adenosyl-L-methionine (SAM) increased the vanillate titer to 272 mg l^−1^. Moreover, the same group harnessed a biosensor for mining highly active methyltransferases, and the vanillate titer was increased to 419 mg l^−1^.

**Fig. 1:**
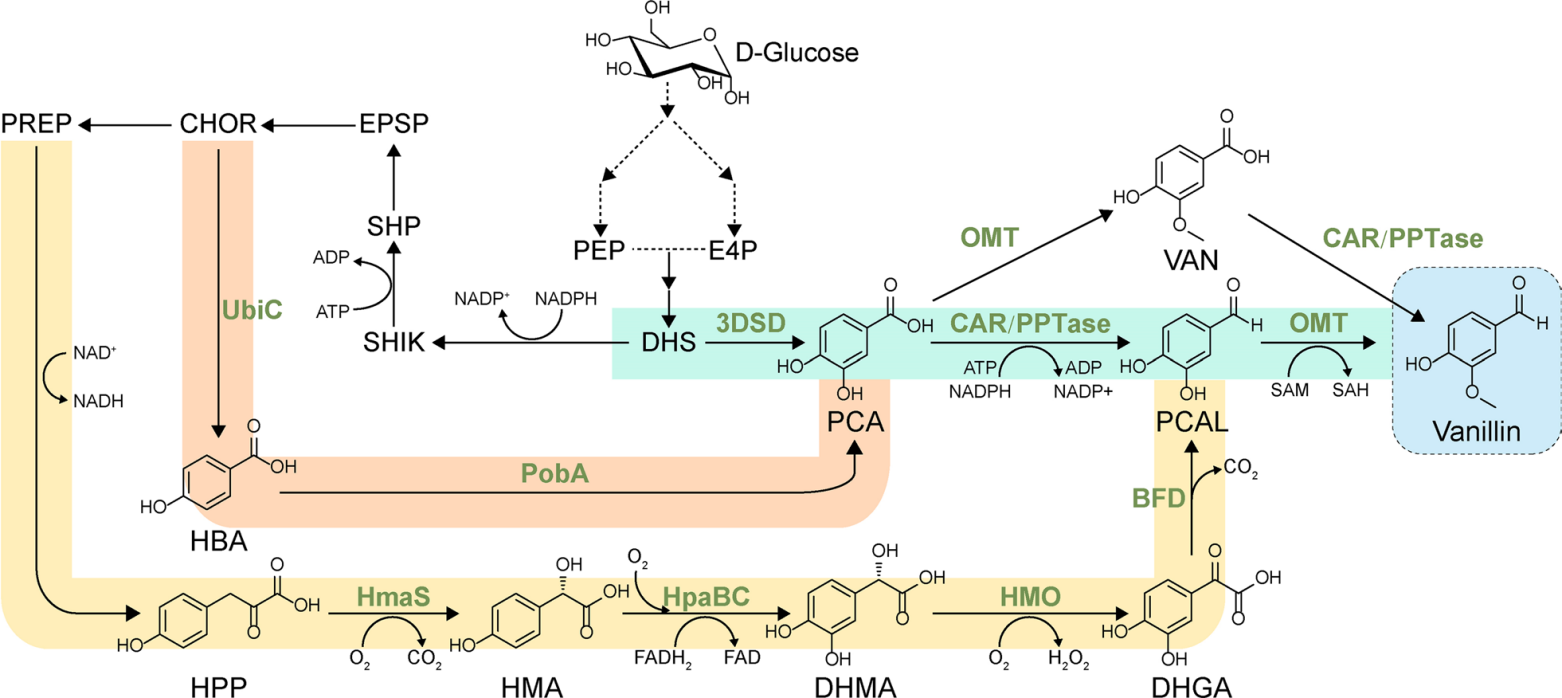
Schematic diagram of de novo synthesis of vanillin in *S. cerevisiae*. Colored boxes represent different metabolic routes towards vanillin synthesis. *3DSD* 3-dehydroshikimate dehydratase, *CAR* carboxylic acid reductase, *PPTase* phosphopantetheine transferase, *OMT* *O*-methyltransferase, *UbiC* chorismate-pyruvate lyase, *PobA* hydroxybenzoate hydroxylase, *HmaS* hydroxymandelate synthase, *HpaBC* two-component flavin-dependent monooxygenase, *HMO* hydroxymandelate oxidase, *BFD* benzoylformate decarboxylase, *PCA* protocatechuate, *PCAL* protocatechualdehyde, *VAN* vanillate, *HBA* hydroxybenzoic acid, *HPP* hydroxyphenylpyruvate, *HMA* hydroxymandelate, *DHMA* 3,4-dihydroxymandelate, *DHGA* 3,4-dihydroxyphenylglyoxylate, *PEP* phosphoenolpyruvate, *E4P* D-erythrose 4-phosphate, *DHS* 3-dehydroshikimate, *SHIK* shikimate, *SHP* shikimate-5-phosphate, *CHOR* chorismate, *EPSP* 5-enolpyruvylshikimate-3-phosphate, *PREP* prephenate

In this study, we investigated a combinatorial deletion strategy to develop a yeast platform with a minimal aromatic aldehyde reduction (MARE). Based on this MARE yeast platform, we implemented multidimensional engineering to increase de novo vanillin synthesis in yeast (Fig. 1). After systematic engineering to optimize the supply of NADPH/SAM, and implementing dual precursor synthetic pathways together with metabolic reconfiguration using a phosphoketolase pathway, the best recombinant strain produced a titer of 365.55 ± 7.42 mg l^−1^ vanillin in shake-flasks, which represents the highest titer from glucose achieved in yeast. Taken together, our work lays a foundation for the future implementation of vanillin production from glucose in budding yeast.

## Results

### Development of a yeast platform for vanillin accumulation

**Fig. 2:**
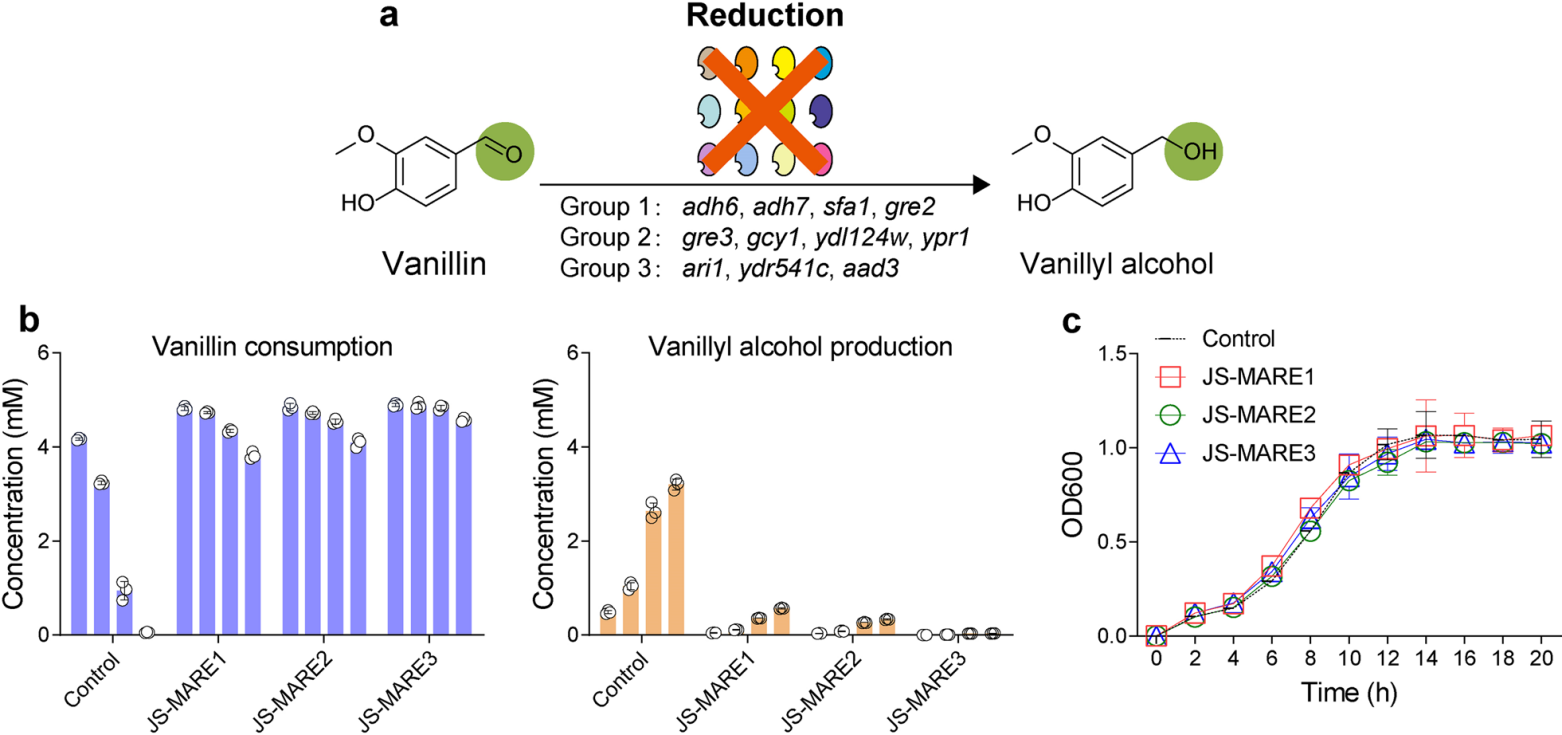
Design and build a minimal aromatic aldehyde reduction (MARE) yeast platform.** a** Schematic diagram showing different sets of oxidoreductases for deletion to reduce vanillin conversion to vanillyl alcohol. **b** The vanillin stability test using engineered *S. cerevisiae.* JS-MARE1 involves the knockout of group 1 oxidoreductases together with ∆*hfd1*:*ubiC*. JS-MARE2 is a JS-MARE1 derivative with the knockout of group 2 oxidoreductases. JS-MARE3 is a JS-MARE2 derivative by further deleting group 3 oxidoreductases. Cells were harvested after 24 h cultivation in SC media. Equal amounts of cells were resuspended into potassium-phosphate buffer (pH 8.0) with 2% glucose + 5 mM of vanillin to a final OD600 of 10. Samples were monitored at regular intervals (4, 8, 24, 48 h) using gas chromatography. **c** The deletion of oxidoreductases did not compromise the growth of engineered yeasts. Control, the parental strain of JS-CR (2 M)

De novo synthesis of vanillin from glucose in *S. cerevisiae* was first reported by Evolva, and 45 mg l^−1^ vanillin was obtained [17]. However, the majority of vanillin was converted to vanillyl alcohol due to the endogenous oxidoreductase activities in budding yeast [17]. Subsequent screening of single knockout of 29 known or hypothesized oxidoreductases revealed that *Adh6* represented the most crucial gene for vanillin reduction, and a 50%-decreased ability of vanillin reduction was achieved by *∆adh6* [17]. More recently, our group addressed the reduction of retinal to retinol in budding yeast by a combined deletion of four ADHs [21], indicating that multiple gene deletion is necessary to improve aldehyde accumulation in yeast [22]. In this study, we implemented the same set of gene deletions (*adh6*, *adh7*, *sfa1* and *gre2*, Fig. 2a), and a dramatic improvement in the vanillin accumulation was observed (Fig. 2b). Notably, the *hfd1* gene was replaced with *ubiC* from *E. coli* to prevent the vanillin oxidation and to compensate the precursor of 4-hydroxybenzoate for ubiquinone synthesis.

Besides ADHs, deletions of additional AKRs and ALDRs in *E. coli* are required to further prevent the aromatic aldehyde reduction [18]. Thus, we proceeded to delete a second set of four genes (*gre3*, *gcy1*, *ydl124w*, and *ypr1*) belongs to AKR family [23] (Fig. 2a). The third set of three genes (*ari1*, *ydr541c*, and *aad3*) related to ALDR family [22] were chosen for deletion (Fig. 2a). The deletion events were confirmed by diagnostic PCR in strain JS-MARE3 (Additional file 1: Figure S1). As shown in Fig. 2b, strain JS-MARE3 had the best performance to prevent vanillin reduction, and vanillyl alcohol was no detectable by gas chromatography even after 48 h. In addition, we found that our engineered yeasts could also improve the accumulation of hydroxybenzaldehyde and PCAL (Additional file 1: Figure S2). Despite the extensive genetic engineering steps, we found that all the engineered strains showed similar growth profiles to the parental strain (Fig. 2c). To the best of our knowledge, our work represented one of the pioneering studies to investigate simultaneous deletion of more than 10 oxidoreductases for rendering the yeast with improved aldehyde-accumulating ability.

### The MARE yeast platform enables the accumulation of de novo synthesized vanillin

Upon the construction of the MARE yeast platform, we next proceeded to investigate whether de novo synthesized vanillin could also be accumulated without forming alcohol byproduct. As shown in Fig. 1, we examined the well-established artificial vanillin biosynthetic pathway that contains 3-dehydroshikimate dehydratase (3DSD) from *Podospora anserina* (encoded by *AroZ* gene) [24], *O*-methyltransferase (OMT) from *Homo sapiens*, and carboxylic acid reductase (CAR) from *Segniliparus rotundus* together with *Nocardia* phosphopantetheine transferase (PPTase) [25]. In brief, 3DSD first converts 3-dehydroshikimate (DHS) into protocatechuate (PCA). Depending on the relative enzyme kinetics and availability of cofactors, PCA can be converted into PCAL or vanillate. The final step in the pathway is either the conversion of PCAL to vanillin by OMT or the vanillate reduction by CAR.

**Fig. 3:**
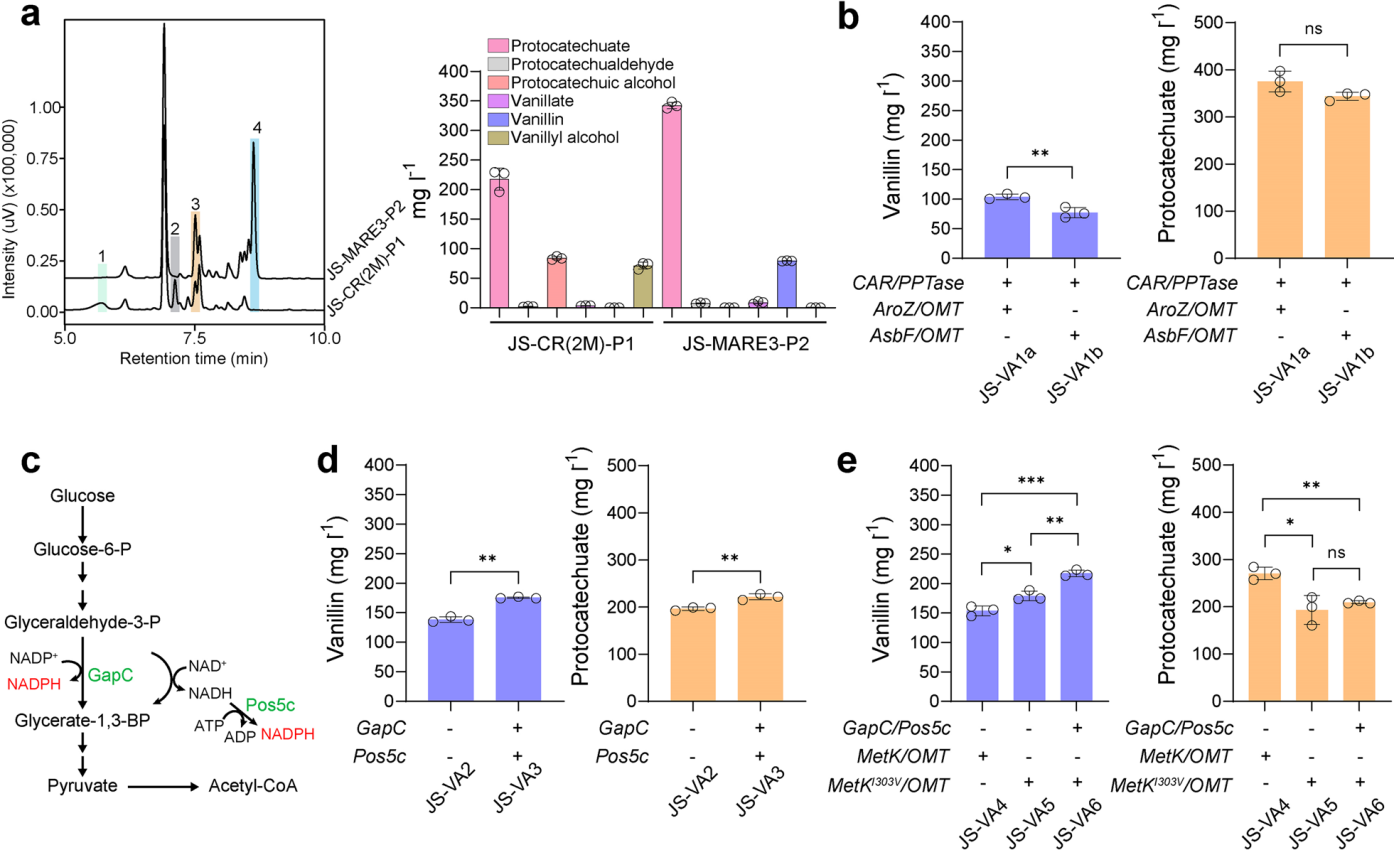
De novo synthesis of vanillin using 3DSD-mediated pathway in *S. cerevisiae*. **a** Representative HPLC results showing vanillin levels in different strains and product distribution profile. Plasmid pRS423-AroZ/OMT and pRS425-CAR/PPTase were transformed into strain JS-CR(2 M) and JS-MARE3, respectively. Peak 1, protocatechuic alcohol; peak 2, vanillyl alcohol; peak 3, PCAL; peak 4, vanillin. The relevant standard curves are shown in Additional file 1: Figure S3. **b** The vanillin and PCA levels in strains with chromosomal 3DSD-mediated pathway. 3DSD encoded by *AroZ* from *P. anserina* and *AsbF* from *B. cereu*s. **c** Schematic illustration of different strategies in improving the NADPH supply. **d** The vanillin and PCA levels in strains with engineered NADPH metabolism. **e** The vanillin and PCA levels in strains with engineered SAM cycle. Cells were grown in SC medium with 2% glucose, and samples were measured after 120 h of cultivation. All experiments were performed in triplicate and the data represent the mean value with standard deviation. Statistical analysis was carried out by using two-tailed unpaired Student’s t-test (*P < 0.05, **P < 0.01, ***P < 0.001)

We first constructed two plasmids, namely, pRS423-AroZ/OMT and pRS425-CAR/PPTase. Based on the plasmid results, the main products produced by the control strain were protocatechuic alcohol and vanillyl alcohol, and only a trace amount of vanillin was accumulated (Fig. 3a). In contrast, JS-MARE3 produced vanillin (79.35 ± 0.39 mg l^−1^) with no detectable amount of vanillyl alcohol (Fig. 3a), confirming that de novo synthesized vanillin is relatively stable in our MARE yeast. Subsequently, we decided to integrate the vanillin biosynthetic modules into the yeast chromosomes for stable genetic inheritance. In particular, AroZ-OMT and CAR-PPTase were integrated at the sites of *rox1* and *bts1*, respectively. As shown in Fig. 3b, chromosomal integration of AroZ-OMT and CAR-PPTase improved the vanillin titer, reaching 104.07 ± 3.74 mg l^−1^ vanillin after 120 h cultivation. However, we observed an accumulation of 375.25 ± 18.12 mg l^−1^ PCA (Fig. 3b), indicating that the CAR-mediated reduction of PCA might be rate-limiting. As CAR might possibly be subjected to a substrate inhibition, we therefore further examined 3DSD from *Bacillus cereu*s (encoded by *AsbF* gene) with a lower activity in yeast. However, the reduced flux of PCA synthesis did not improve vanillin synthesis, and the strain with AsbF from *B. cereus* only resulted in 77.17 ± 7.24 mg l^−1^ vanillin after 120 h (Fig. 3b).

To further improve the CAR activity for vanillin synthesis, we attempted to integrate an additional copy of PPTase from difference sources (*Sfp* from *Bacillus subtilis* and *EntD* from *E. coli*) [17] at the *ypl062w* site of JS-VA1a, the resulting strain JS-VA2 increased the vanillin titer to 138.50 ± 3.81 mg l^−1^ (Additional file 1: Figure S4). However, the total amount of intermediates such as PCA, vanillate and PCAL was reduced (Additional file 1: Figure S4), indicating that disruption of *ypl062w* might affect the overall productivity of 3DSD-mediated pathway. According to the literature, the ypl062w mutant was previously shown to have lower levels of glycogen [26]. In this mutant, less carbon is sequestered in the form of glycogen, making more acetyl-coA available for other metabolic pathway [27]. It seems that ypl062w has a certain role in allowing the rapid formation of acetyl-CoA, which is not favorable for PCA synthesis.

### Engineering the cofactor supply for improving vanillin synthesis

Considering the 3DSD-mediated vanillin biosynthetic pathway is limited because the CAR activity is not optimal in yeast, we next proceeded to optimize the abundance of cofactors (NADPH and ATP) for improving the CAR step. As shown in Fig. 3c, NADPH-dependent glyceraldehyde-3-phosphate dehydrogenase (GapC) from *Clostridium acetobutylicum* [28] and a cytosol-relocalized NADH kinase (Pos5c) [29] from *S. cerevisiae* were introduced to improve the NADPH supply, and the resulting strain was designated as JS-VA3. Upon overexpressing GapC and Pos5c, strain JS-VA3 produced 175.29 ± 1.16 mg l^−1^ vanillin and 221.66 ± 5.12 mg l^−1^ PCA (Fig. 3d). In addition, the intermediate vanillate was reduced from 20.01 ± 1.26 mg l^−1^ to 9.32 ± 0.92 mg l^−1^ (Additional file 1: Figure S5). Although overexpressing GapC and Pos5c could increase the vanillin production, it still did not completely solve the problem of PCA accumulation.

Next, we further proceeded to engineering the SAM supply cycle to improve the vanillin synthesis. The accumulation of PCA instead of vanillate suggested that the activity of OMT from *H. sapiens* for the methylation of PCA was clearly insufficient. The *E. coli* SAM synthetase (MetK with a mutation of I303V) was previously reported to have a fourfold increase in activity with decreased product inhibition [30]. By introducing the genes *MetK* or *MetK*^*I303V*^ together with an additional copy of *OMT* into the *yjl064w* site of strain JS-VA2, we found that the resulting strain JS-VA4 and JS-VA5 produced 153.74 ± 6.75 mg l^−1^ and 179.04 ± 6.82 mg l^−1^ vanillin, respectively (Fig. 3e). Furthermore, we have observed that the level of vanillate is lower in the MetK^I303V^ mutant strain compared to the WT MetK strain (Additional file 1: Figure S5). This can be attributed to the conversion of vanillate into vanillin, which subsequently reduces the vanillate content. Interestingly, we have also observed a significant decrease in the PCA content in JS-VA5 compared to JS-VA4 (Fig. 3e). These findings further supported that MetK^I303V^ has a stronger ability to methylate PCA into vanillate, and the conversion of PCA to vanillate is more efficient in the JS-VA5 strain. Further combined with GapC-Pos5c into JS-VA5 resulted in additional 21.36% improvement of vanillin in JS-VA6 (Fig. 3e), achieving 217.29 ± 4.65 mg l^−1^ vanillin after 120 h cultivation. The vanillate was reduced to 209.40 ± 2.66 mg l^−1^ in JS-VA6 (Additional file 1: Figure S5), whereas no significant difference of PCA was observed in JS-VA6 (Fig. 3e). Taken together, these results proved that the activation of SAM cycle by overexpressing MetK^I303V^ can promote the methylation process mediated by OMT.

### Dual precursor synthetic pathways to improve vanillin production

**Fig. 4:**
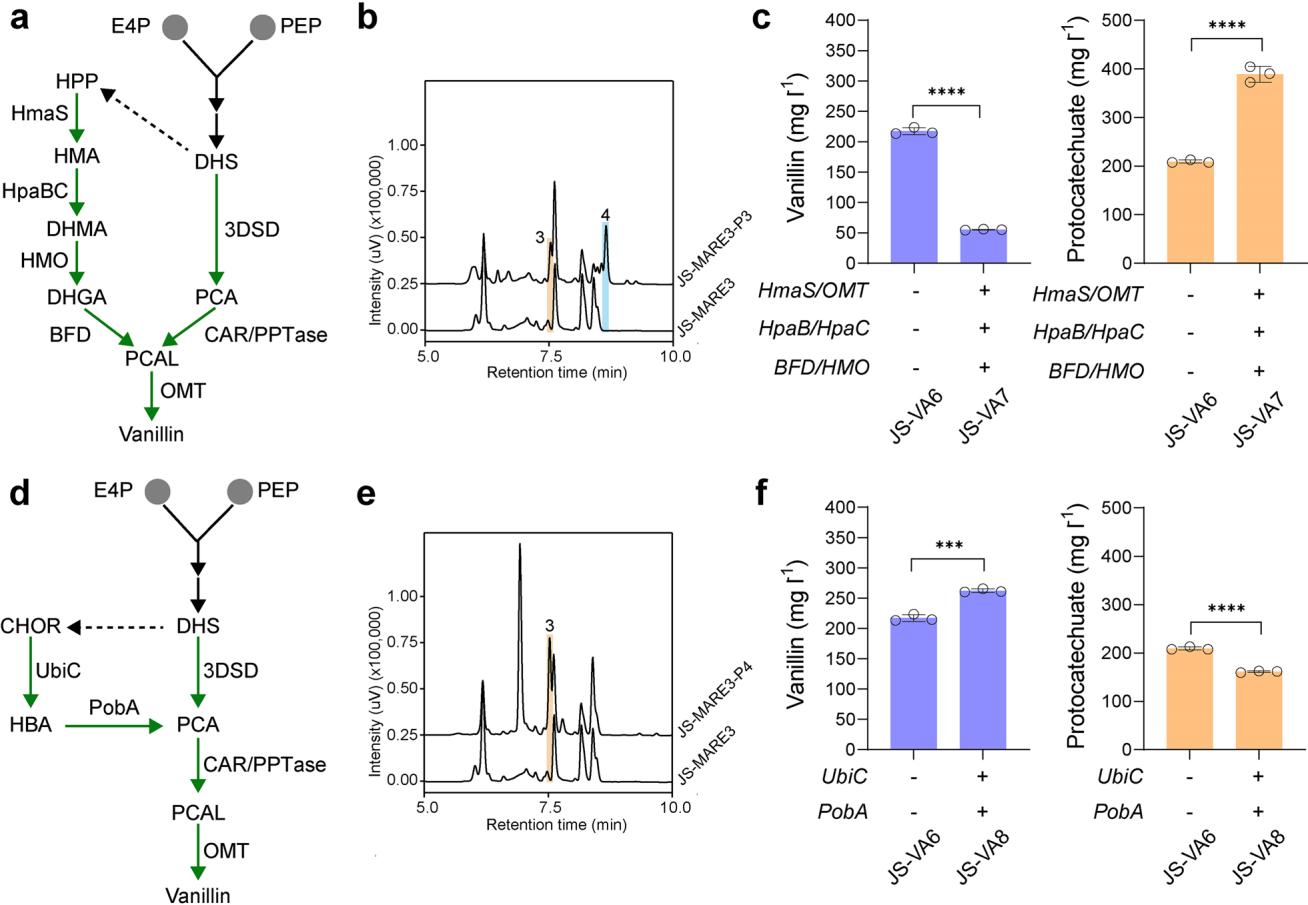
Dual synthetic pathway for enhanced vanillin synthesis in yeast.** a** Schematic of HmaS-mediated pathway for synthesizing vanillin. **b** Representative HPLC results of HmaS-mediated pathway for vanillin synthesis. Strains harboring plasmids with HmaS-OMT-HpaBC-HMO-BFD were used. **c**, The vanillin and PCA levels in strains harboring dual 3DSD and HmaS-mediated vanillin pathway. **d** Schematic of UbiC-PobA pathway for synthesizing vanillin.** e** Representative HPLC results of UbiC-PobA coupled with CAR-PPTase for PCAL synthesis. Strains harboring plasmids with UbiC-PobA-CAR-PPTase were used. **f** The vanillin and PCA levels in strains harboring dual 3DSD and UbiC-PobA vanillin pathway. Cells were grown in SC medium with 2% glucose, and samples were measured after 120 h of cultivation. All experiments were performed in triplicate and the data represent the mean value with standard deviation. Statistical analysis was carried out by using two-tailed unpaired Student’s *t*-test (***P < 0.001, ****P < 0.0001)

Recently, our group have demonstrated that two-component flavin-dependent monooxygenase (HpaBC) from *E. coli*, hydroxymandelate synthase (HmaS) from *Amycolatopsis orientalis*, hydroxymandelate oxidase (HMO) from *Streptomyces coelicolor* and benzoylformate decarboxylase (BFD) from *Pseudomonas putida* allowed the synthesis of PCAL [31]. To further harness the metabolic flux from L-tyrosine synthesis, we transplanted this coenzyme A independent pathway into yeast for vanillin synthesis (Fig. 4a). As the HpaBC pair containing HpaB from *Pseudomonas aeruginosa* and HpaC from *Salmonella enterica* had a better activity when heterologously expressed in *S. cerevisiae* [32], we therefore replaced HpaBC from *E. coli* with PaHpaB-SeHpaC. Subsequently, we integrated HmaS-OMT at the *pha2* locus to restrict L-phenylalanine synthesis, BFD-HMO at the *are1* locus to minimize ergosteryl ester levels [33], and PaHpaB-SeHpaC at the *gdh1* locus to further reduce NADPH consumption [34].

We first examined plasmid-based expression of HmaS-OMT-HpaBC-HMO-BFD for vanillin production. It was found that HmaS-mediated synthetic vanillin pathway functioned in yeast based on the HPLC results (Fig. 4b), reaching up to 19.81 ± 1.54 mg l^−1^ vanillin under shake-flask cultivation (Additional file 1: Figure S6). However, the engineered strain with dual 3DSD and HmaS-mediated vanillin pathway (strain JS-VA7) only produce 55.19 ± 0.60 mg l^−1^ vanillin, and more than 1.8-fold of PCA was accumulated over that of the parental strain JS-VA6 (Fig. 4c). These findings indicated that the intermediates from HmaS-mediated pathway might inhibit the CAR activity. When strain JS-VA6 was supplemented with an additional 1 mM hydroxymandelate, the vanillin level was reduced from 53.31 ± 1.54 mg l^−1^ to 26.41 ± 1.40 mg l^−1^ in small-scale shake tubes for 48 h (Additional file 1: Figure S7), suggesting that the CAR activity might be competitively inhibited by hydroxymandelate analogs. Overall, HmaS-mediated biosynthetic pathway was functional but not compatible with the CAR-mediated vanillin pathway.

Alternatively, we also investigated chorismate pyruvate lyase (UbiC from *E. coli*) [35] coupled with hydroxybenzoate hydroxylase (PobA from *P. putida*) [36] for vanillin synthesis (Fig. 4d). Based on the plasmid results, PCAL was successfully produced upon introducing UbiC-PobA-CAR-PPTase (Fig. 4e). As shown in Fig. 4f, further integration of UbiC-PobA at the *gdh1* locus resulted in 262.27 ± 2.36 mg l^−1^ vanillin in strain JS-VA8, which represents 20.70% improvement over that of strain JS-VA6. Surprisingly, the PCA level in JS-VA8 was reduced to 161.29 ± 1.40 mg l^−1^ when compared to that of JS-VA6 (Fig. 4f), whereas there was no significant difference in the vanillate level (Additional file 1: Figure S8). Since the UbiC can split chorismate to give a pyruvate molecule, it might provide more ATP/NADPH for boosting the CAR activity. In addition, the perturbation of enzyme levels might also explain the discrepancy of reduced PCA in JS-VA8. These findings suggested that the intricate self-regulatory mechanisms within the yeast cell have to be carefully evaluated when multiple pathways are introduced for producing a targeted product.

### Phosphoketolase pathway to improve the supply of E4P precursor to improve vanillin production

**Fig. 5:**
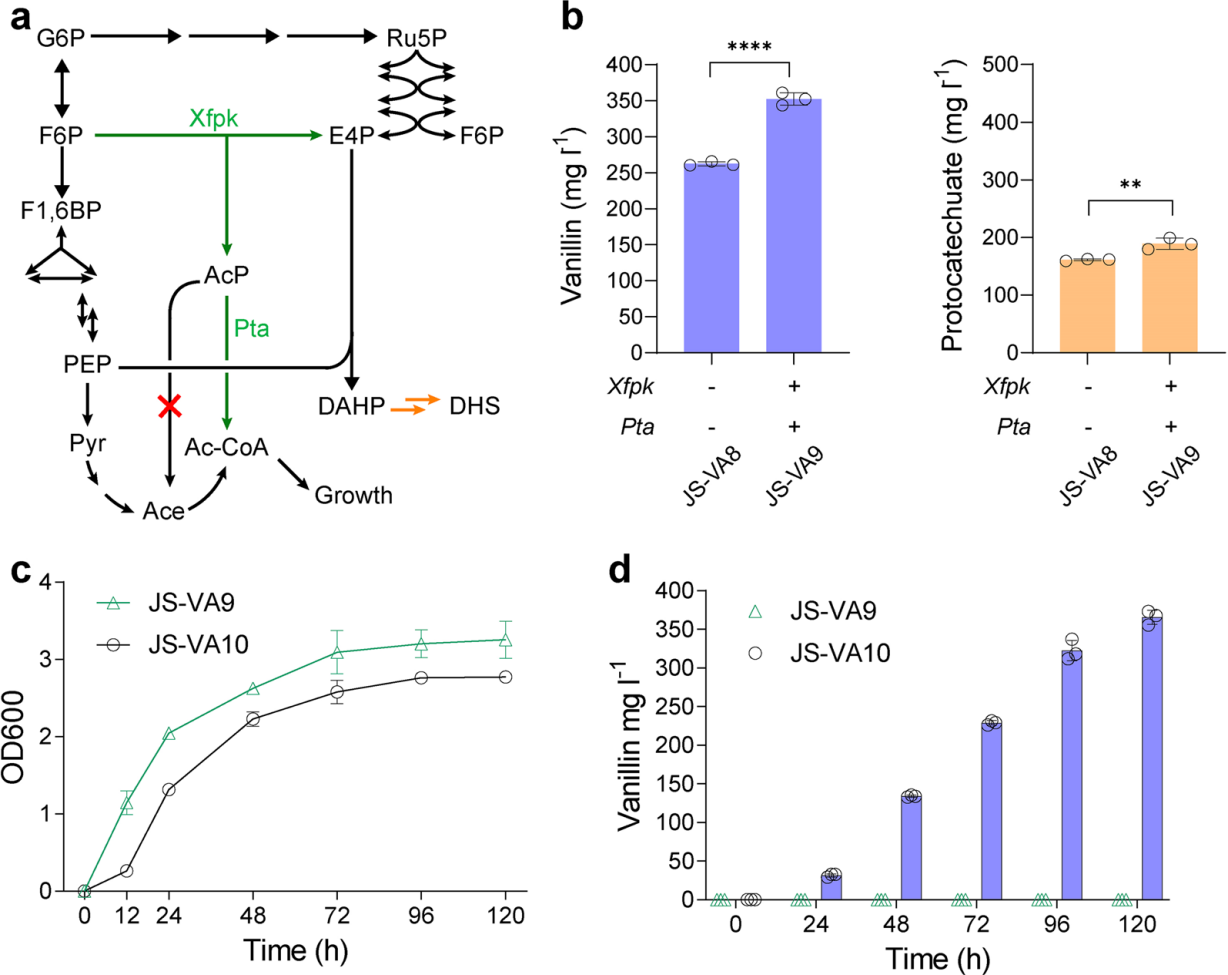
Metabolic reconfiguration for further enhanced vanillin production.** a** Schematic overview of Xfpk-Pta pathway for improving the precursor supply of E4P. **b** The effect of Xfpk-Pta pathway on the vanillin and PCA productions. The glycerol-1-phosphatase GPP1 was deleted to minimize acetate formation (marked with a red cross). Cells were grown in SC medium with 2% glucose, and samples were measured after 120 h of cultivation. **c** The growth curve of strain JS-VA9 and JS-VA10 in shake-flasks. Strain JS-VA10 was a derivative of JS-VA9 with Ubi-K15N degron fused to the N-terminal of Gal80. Both strains were cultivated in YPD medium containing 20 g l^−1^ glucose. **d** Vanillin produced by strain JS-VA9 and JS-VA10 in YPD media. All experiments were performed in triplicate and the data represent the mean value with standard deviation. Statistical analysis was carried out by using two-tailed unpaired Student’s t-test (**P < 0.01, ****P < 0.0001)

To further enhance the performance of vanillin production, we also attempted to reconfigure the yeast central metabolism for an improved precursor supply of D‐erythrose 4‐phosphate (E4P). Recently, a phosphoketalose-based pathway (Xfpk-Pta) by providing more E4P was proven to be effective in increasing aromatic chemical productions (Fig. 5a) [37, 38]. In addition, acetyl-CoA from Xfpk-Pta pathway could be used for ATP generation, which also favorably drives the CAR step. As shown in Fig. 5b, the vanillin titer in strain JS-VA9 was further increased by 34.32% upon introducing a phosphoketolase from *Bifidobacterium breve* (BbXfpk) and a phosphotransacetylase from *Clostridium kluyveri* (CkPta), reaching 352.28 ± 7.03 mg l^−1^ vanillin (17.61 ± 0.35 mg vanillin per g glucose) in shake-flasks.

To evaluate the performance of the engineered strain in yeast-peptone-dextrose (YPD) medium, we further engineered the copper-inducible system with Ubi-K15N tagged Gal80 [39], to reduce the half-life of Gal80. As shown in Fig. 5c, the OD600 of JS-VA10 was lower than that of the control strain JS-VA9 (no vanillin production in YPD medium). Strain JS-VA10 resulted in 365.55 ± 7.42 mg l^−1^ vanillin after 120 h cultivation in the YPD medium (Fig. 5d). In the future, cell-density dependent system might be further explored for auto-production of vanillin in yeast [40]. Based on our toxicity assay as shown in Additional file 1: Figure S9, the yeast cells can tolerate < 1 g l^−1^ vanillin and multiple genetic modifications of oxidoreductase deletions did not change the yeast tolerance to vanillin. However, the titer of vanillin in YPD was only slightly improved over that in SC medium, indicating that de novo synthesized vanillin might be even more toxic than extracellularly supplemented vanillin. Therefore, the bottleneck for industrial production of vanillin would be the toxicity of vanillin to the cells [17] and future industrial-scale vanillin production in yeast would require strain engineering to improve the yeast tolerance to vanillin.

## Discussion

In this study, we have constructed a MARE yeast platform that accumulates an industrially relevant aromatic aldehyde of vanillin. The optimal aldehyde-accumulating result was achieved by combined deletion of 11 genes, spanning the ADH, AKR, and ALDR super-families. Despite all these deletions, the growth rate of the engineered strains remained nearly uncompromised. Besides, we also observed that a variety of aromatic aldehydes (hydroxybenzaldehyde, protocatechualdehyde) could also be accumulated in the engineered yeast, suggesting that the MARE yeast platform might be applicable for future synthesis of many aldehyde-derived compounds.

Natural vanillin biosynthetic pathways typically reply on ferulic acid as the precursor, which is converted to vanillin by a single hydratase/lyase-type enzyme designated as VpVAN, or by a CoA-dependent pathway comprising feruloyl-CoA synthetase (FCS) and enoyl-CoA hydratase/aldolase (ECH) [16]. In budding yeast, the endogenous FDC1 and PAD1 could rapidly decarboxylate phenylacrylic acids such as ferulic acid, caffeic acid and coumaric acid [41]. In this study, we used artificial vanillin biosynthetic pathways to bypass the intermediate of ferulic acid. In particular, we first assembled a synthetic pathway comprising 3DSD, OMT, CAR, and PPTase. We found that *AroZ* gene from *P. anserina* was more effective for PCA production than *AsbF* gene from *B. cereus*. In addition, we noticed the intracellular SAM was insufficient even after overexpressing MetK^I303V^, indicating that further engineering the SAM cycle to improve the SAM supply is still required to address the vanillin productivity at the OMT-mediated methylation step [32]. Although we attempted to improve the CAR-step by increasing the abundance of coenzymes and cofactors (NADPH and ATP), the primary limiting factor for 3DSD-mediated vanillin synthesis seems to be the insufficient activities of CAR, which resulted in a high amount accumulation of PCA. Considering CAR from *Mycobacterium abscessus* was recently reported to have a relatively high activity for the vanillate reduction [42], this alternative version of CAR might potentially solve the bottleneck of 3DSD-mediated vanillin production. In addition, the methylation step in yeast was also rate-limiting, which requires an extensive study to improve the vanillin synthesis.

It was found that the heterologous hydroxymandelate degradation pathway [43, 44] was functional in budding yeast, but this pathway was not compatible with 3DSD-mediated vanillin synthesis as the CAR activity would be repressed by hydroxymandelate analogs. In the future, it will be interesting to see whether a CoA-dependent pathway comprising FCS/ECH might be coupled with HmaS to give a better vanillin titer. Noteworthily, introducing UbiC-PobA further improved the vanillin titer in the strain equipped with 3DSD-mediated vanillin pathway and the synthetic yeast factory with dual vanillin biosynthetic pathway produced 262.27 ± 2.35 mg l^−1^ vanillin in shake-flasks. Further introducing Xfpk from *B. breve* and Pta from *C. kluyveri* to improve the precursor E4P supply enabled 352.28 ± 7.03 mg l^−1^ vanillin in shake-flasks, which represents the highest vanillin titer from glucose.

According to the literature, nonglucosylated vanillin beyond the 0.5 ~ 1 g l^−1^ scale would severely hamper the growth of *S. cerevisiae* [17]. The remaining bottleneck for vanillin overproduction in yeast is the intrinsic toxicity of vanillin to the host cells. Adaptive laboratory evolution (ALE) has proven a useful strategy to acquire desired phenotypes with accumulation of beneficial mutations under selective pressure. Moreover, global transcription machinery engineering (gTME) by mutagenesis of the transcription factor could lead to dominant mutations that confer increased tolerance and more efficient glucose conversion to ethanol [45]. In the future, ALE and gTME to reprogram the global cell metabolism might be implemented to improve the vanillin tolerance in budding yeast. In addition, diploid *S. cerevisiae* or other yeast species with a better tolerance might be used as the starting chassis for industrial-scale vanillin production. Alternatively, in situ vanillin recovery using resin with high selectivity and loading capacity might be used to address the toxicity issue of vanillin. Therefore, we believe that sustainable production of natural vanillin from glucose would be eventually achieved by continuous efforts in metabolic engineering, synthetic biology and process optimization.

## Materials and methods

### Strains, culture media and reagents

*S. cerevisiae* BY4741 derived JS-CR(2 M) with the galactose regulon under the copper-inducible system [46] was used as the initial chassis for constructing all the subsequent strains. The YPD medium (10 g l^−1^ yeast extract, 20 g l^−1^ peptone and 20 g l^−1^ glucose) was used for cultivation of yeast cells, and synthetic complete (SC) medium with appropriate dropouts was used for yeast cells with different auxotrophic markers. *E. coli* DH5α was used as the recipient strain for cloning plasmids, and the strains carrying the plasmid were cultured at 37 °C in Luria–Bertani broth with 100 μg ml^−1^ ampicillin. All restriction enzymes, T4 ligase, Taq polymerase and High-fidelity Phusion polymerase were obtained from New England Biolabs (Beverly, MA, USA). Gel extraction kit and plasmid purification kit were purchased from BioFlux (Shanghai, China). Antibiotics, 5-fluoroorotic acid and oligonucleotides were purchased from Sangon Biotech (Shanghai, China). The standard vanillin (Cat. No. V100115), vanillyl alcohol (Cat. No. H103777), vanillate (Cat. No. V104428), hydroxybenzaldehyde (Cat. No. H100420), hydroxybenzyl alcohol Cat. No. H107912), protocatechualdehyde (Cat. No. D108634), protocatechuic alcohol (Cat. No. D155345), and protocatechuate (Cat. No. P104382) were purchased from Aladdin Biotech (Shanghai, China). All the other chemicals were obtained from Sigma-Aldrich or otherwise stated.

### Plasmid construction

Oligonucleotides used for plasmid construction are listed in Additional file 1: Table S1. *HmaS* from *A. orientalis*, *OMT* from *H. sapiens*, *HpaB* from *P. aeruginosa*, *Xfpk* from *B. breve* and *Pta* from *C. kluyveri* were synthesized by GenScript (Additional file 1: Table S2). *MetK*, *UbiC* and *EntD* were PCR amplified from the genomic DNA of *E. coli* MG1655. *HpaC* was PCR amplified from the genomic DNA of *S. enterica* LT2. *HMO* was PCR amplified from the genomic DNA of *S. coelicolor* A3(2). *Segniliparus CAR* and *Nocardia PPTase* were kindly provided by Prof. Dunming Zhu from Tianjin Institute of Industrial Biotechnology, Chinese Academy of Sciences. *AroZ* was a gift from Prof. Eckhard Boles from Goethe University Frankfurt. *BFD* and *PobA* were PCR amplified from the genomic DNA of *P. putida* KT2440. *Sfp* and *AsbF* was PCR amplified from the genomic DNA of *B. subtilis* 168. *Pos5c* was PCR amplified from the genomic DNA of *S. cerevisiae* BY4741. *GapC* was PCR amplified from the genomic DNA of *C. acetobutylicum* ATCC 824. Plasmid pRS423-AroZ/OMT, pRS423-AsbF/OMT, pRS425-CAR/PPTase, pRS423-HpaB/HpaC, pRS425-BFD/HMO, pRS426-HmaS/OMT, pRS423-MetK/OMT, pRS423-MetK^I303V^/OMT, pRS423-Xfpk/Pta, pRS426-UbiC/PobA, and pRS425-Sfp/EntD, were all constructed via the golden-gate approach [47]. All the plasmids used in this study are provided in Additional file 1: Table S3.

### Genome editing of *S. cerevisiae*

The CRISPR/Cas9 genome editing was carried out as previously described [48]. The guide RNA (gRNA) expression plasmids were derived from an in-house plasmid of pRS426SNR52. The standard protocol of *S. cerevisiae* transformation was carried out by electroporation with minimal modification. 50 μl of yeast cells together with approximately 2 μg mixture of genome editing cassette was electroporated in a 0.2 cm cuvette at 1.6 kV. After electroporation, cells were immediately mixed with 900 μl YPD medium and recovered a rotary shaker for 1 h. Cells were plated on SC plate with appropriate dropouts. Successful genome manipulations were confirmed by diagnostic PCR before proceeding to the next round of genetic modifications. Subsequently, gRNA expressing plasmid was eliminated via counter-selection with 1 g l^−1^ 5-fluoroorotic acid (5-FOA), and the Cas9-expressing plasmid was removed via a series dilution. The flowchart of yeast strain construction is provided in Additional file 1: Figure S10. All the strains used in this study are provided in Additional file 1: Table S4.

### Aldehyde depletion assay in *S. cerevisiae*

The engineered *S. cerevisiae* strains were harvested after 24 h cultivation in SC media. Equal amounts of cells were resuspended into potassium-phosphate buffer (pH 8.0) with 2% glucose + 5 mM of vanillin to a final OD600 of 10. Samples were monitored at regular intervals (4, 8, 24, 48 h) using gas chromatography. The depletion assay for hydroxybenzaldehyde and PCAL were carried out in a similar way as mentioned-above.

### Shake-flask cultivation for vanillin production

For small-scale production of vanillin, experiments were carried out using 100-ml shake-flasks. 1% fresh overnight culture was inoculated into shake-flasks containing 10 ml SC medium supplemented with 20 μM copper sulfate. The cultures were incubated at 30 °C and 250 rpm for vanillin productions. For gas chromatography-flame ionization detector (GC-FID) analysis of vanillin, 100 μl of supernatant was extraction with 900 μl ethyl acetate before subjected to GC-FID analysis. 1 μl of diluted sample was injected into GC-2030 system equipped with an Rtx-5 column (30 m × 250 μm × 0.25 μm thickness). Nitrogen (ultra-purity) was used as carrier gas at a flow rate 1.0 ml min^−1^. GC oven temperature was initially held at 40 °C for 2 min, increased to 45 °C with a gradient of 5 °C min^−1^ and held for 4 min. And then it was increased to 230 °C with a gradient 15 °C min^−1^.

For high-performance liquid chromatography (HPLC) analysis of vanillin, Shimadzu Prominence LC-20A system (Shimadzu, Japan) equipped with a reversed phase C18 column (150 × 4.6 mm, 2.7 μm) and a photodiode array detector was used. The samples were centrifuged and filtered through a 0.2-μm syringe filter before injected to the HPLC system. The mobile phase comprises solvent A (ddH_2_O with 0.1% trifluoroacetic acid) and solvent B (acetonitrile with 0.1% trifluoroacetic acid). The following gradient elution was used: 0 min, 95% solvent A + 5% solvent B; 8 min, 20% solvent A + 80% solvent B; 10 min, 80% solvent A + 20% solvent B; 11 min, 95% solvent A + 5% solvent B. The flow rate was set at 1 ml min^−1^. The levels of vanillin and other aromatic compounds were monitored at the absorbance of 275 nm.

### Analysis of the growth-inhibitory effect of vanillin on yeast

Fresh overnight cultures of yeast strains were inoculated into SC media supplemented with different concentrations of vanillin (0.25, 0.5, 0.75, 1.0, and 1.5 g l^−1^), whereas no additional vanillin supplementation was used as the control. The yeast cultures were then grown at 30 °C on a rotary shaking incubator at 250 rpm. The OD600 was measured with regular time intervals (4, 8, 12, 16, 20, 24, and 28 h).

### Supplementary Information

**Additional file 1: Table S1.** Oligonucleotides used in this study. **Table S2.** Synthesized genes used in this study. **Table S3.** Plasmids used in this study. **Table S4.** Strains used in this study**. Figure S1.** Knockout of the endogenous oxidoreductases in budding yeast. **Figure S2.** Other aromatic aldehyde accumulation in the MARE yeast. **Figure S3.** The standard curves of authentic compounds. **Figure S4.** The effect of Sfp and EntD expression on vanillin production. **Figure S5.** Product profiles of protocatechualdehyde and vanillate produced by engineered yeasts of JS-VA2~6. **Figure S6.** *De novo* synthesis of vanillin from plasmid-based HmaS pathway in *S. cerevisiae*. **Figure S7.** The inhibitory effect of hydroxymandelate to the CAR-mediated vanillin biosynthetic pathway. F**igure S8.** Product profiles of protocatechualdehyde and vanillate produced by engineered yeasts of JS-VA6~9. **Figure S9.** The growth inhibitory effect of vanillin to *S. cerevisiae*. **Figure S10.** Flowchart of yeast strain construction in this study.

## Data Availability

All data generated or analyzed during this study are included in this published article and its Additional files.
