# Supplementary material for: Minimal aromatic aldehyde reduction (MARE) yeast platform for engineering vanillin production
Source: Biotechnol Biofuels Bioprod. 2024 Jan 6;17:4. doi: 10.1186/s13068-023-02454-5 (PMC10771647; doi:10.1186/s13068-023-02454-5)
Supplement: Supplementary file 1 — Additional file 1: Table S1. Oligonucleotides used in this study. Table S2. Synthesized genes used in this study. Table S3. Plasmids used in this study. Table S4. Strains used in this study. Figure S1. Knockout of the endogenous oxidoreductases in budding yeast. Figure S2. Other aromatic aldehyde accumulation in the MARE yeast. Figure S3. The standard curves of authentic compounds. Figure S4. The effect of Sfp and EntD expression on vanillin production. Figure S5. Product profiles of protocatechualdehyde and vanillate produced by engineered yeasts of JS-VA2~6. Figure S6. De novo synthesis of vanillin from plasmid-based HmaS pathway in S. cerevisiae. Figure S7. The inhibitory effect of hydroxymandelate to the CAR-mediated vanillin biosynthetic pathway. Figure S8. Product profiles of protocatechualdehyde and vanillate produced by engineered yeasts of JS-VA6~9. Figure S9. The growth inhibitory effect of vanillin to S. cerevisiae. Figure S10. Flowchart of yeast strain construction in this study. [file 13068_2023_2454_MOESM1_ESM.docx]

**Supplementary information**

**Minimal Aromatic Aldehyde Reduction (MARE) yeast platform for engineering vanillin production**

Qiwen Mo^1^ and Jifeng Yuan^1,^*

^1^ State Key Laboratory of Cellular Stress Biology, School of Life Sciences, Faculty of Medicine and Life Sciences, Xiamen University, Fujian, China 361102

* Corresponding author address: State Key Laboratory of Cellular Stress Biology, School of Life Sciences, Faculty of Medicine and Life Sciences, Xiamen University, Fujian, China 361102. Email address: jfyuan@xmu.edu.cn

**Supplementary Figures**

**
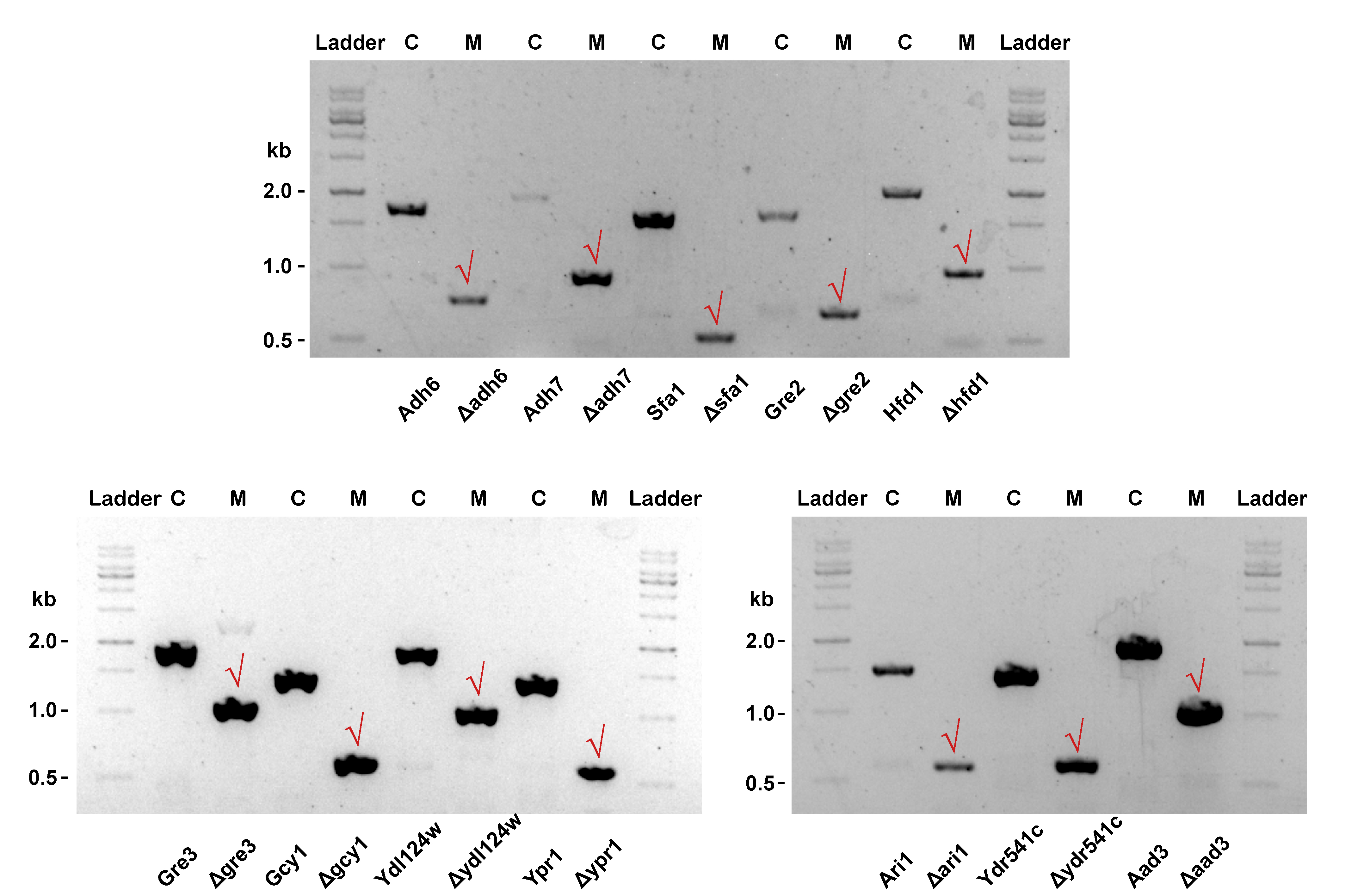
**

**Figure S1. Knockout of the endogenous oxidoreductases in budding yeast.** C, control; M, JS-MARE3.


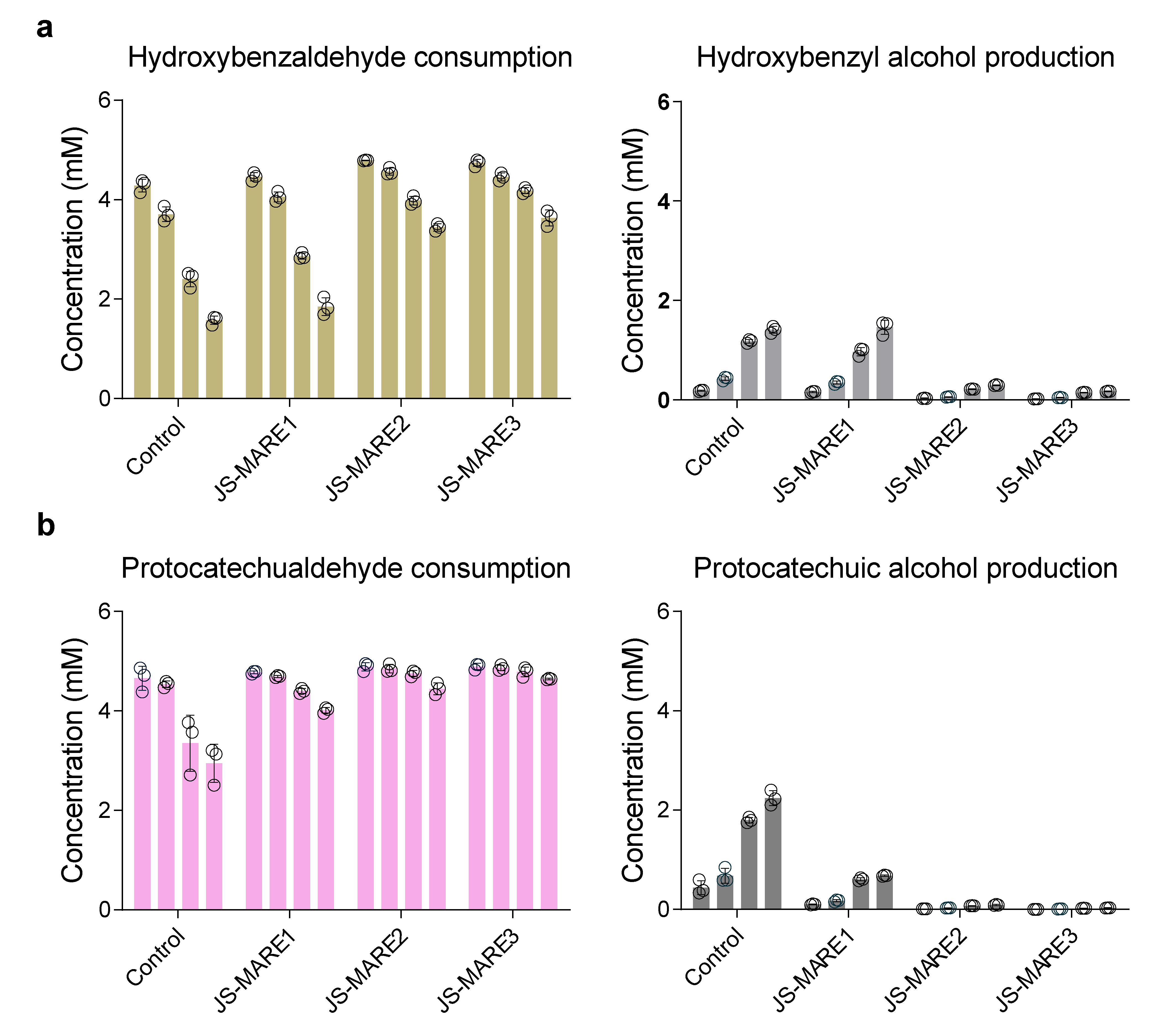


**Figure S2. Other aromatic aldehyde accumulation in the MARE yeast.** **a,** Hydroxybenzaldehyde stability test using the engineered *S. cerevisiae*. **b,** Protocatechualdehyde stability test using the engineered *S. cerevisiae*. Cells were harvested after 24 h cultivation in SC media. Equal amounts of cells were resuspended into KP buffer (pH 8.0) with 2% glucose + 5 mM of hydroxybenzaldehyde or protocatechualdehyde to a final OD600 of 10. Samples were periodically monitored by HPLC or GC-FID analysis for 4, 8, 24, 48 h.

**
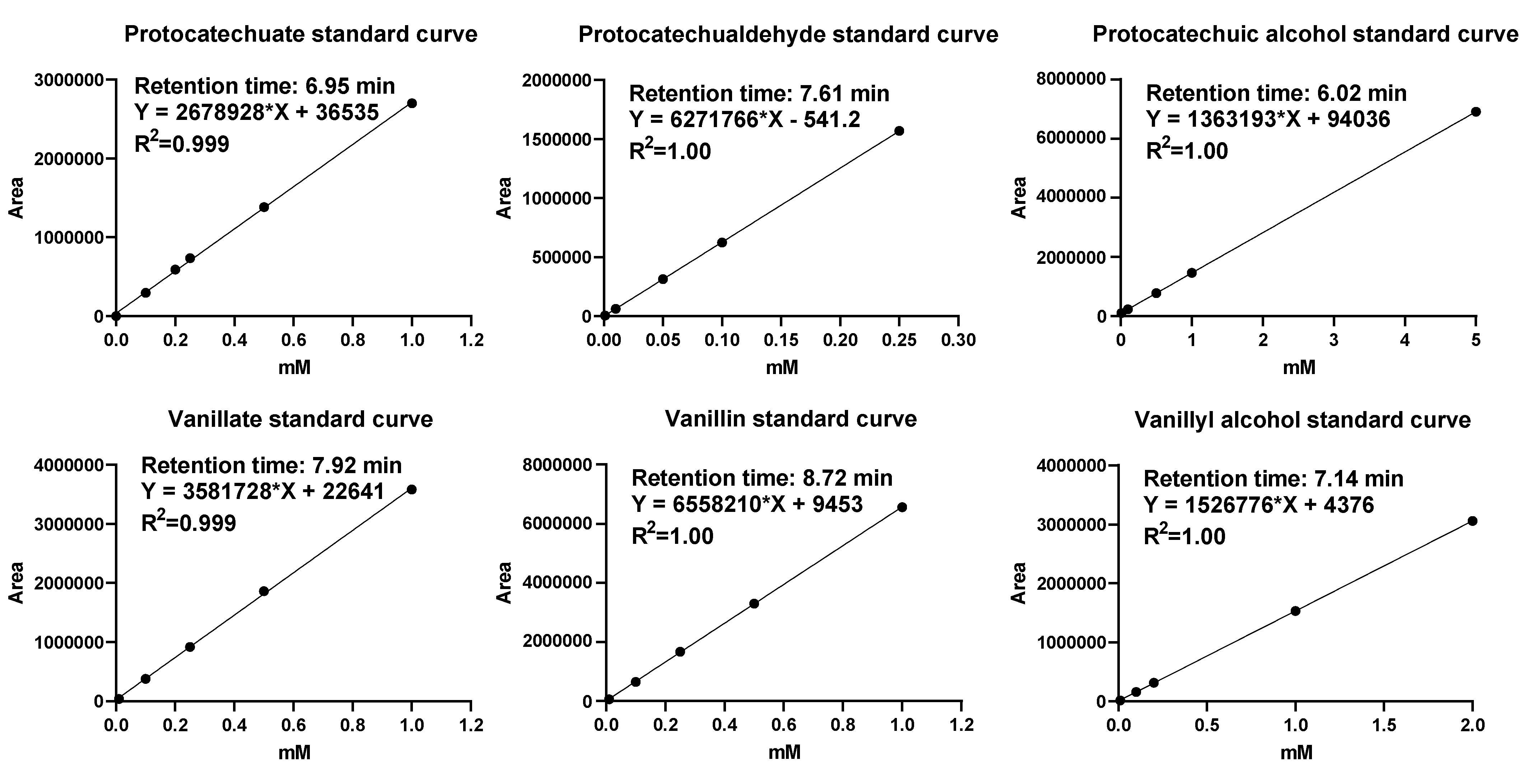
**

**Figure S3. The standard curves of authentic compounds.**


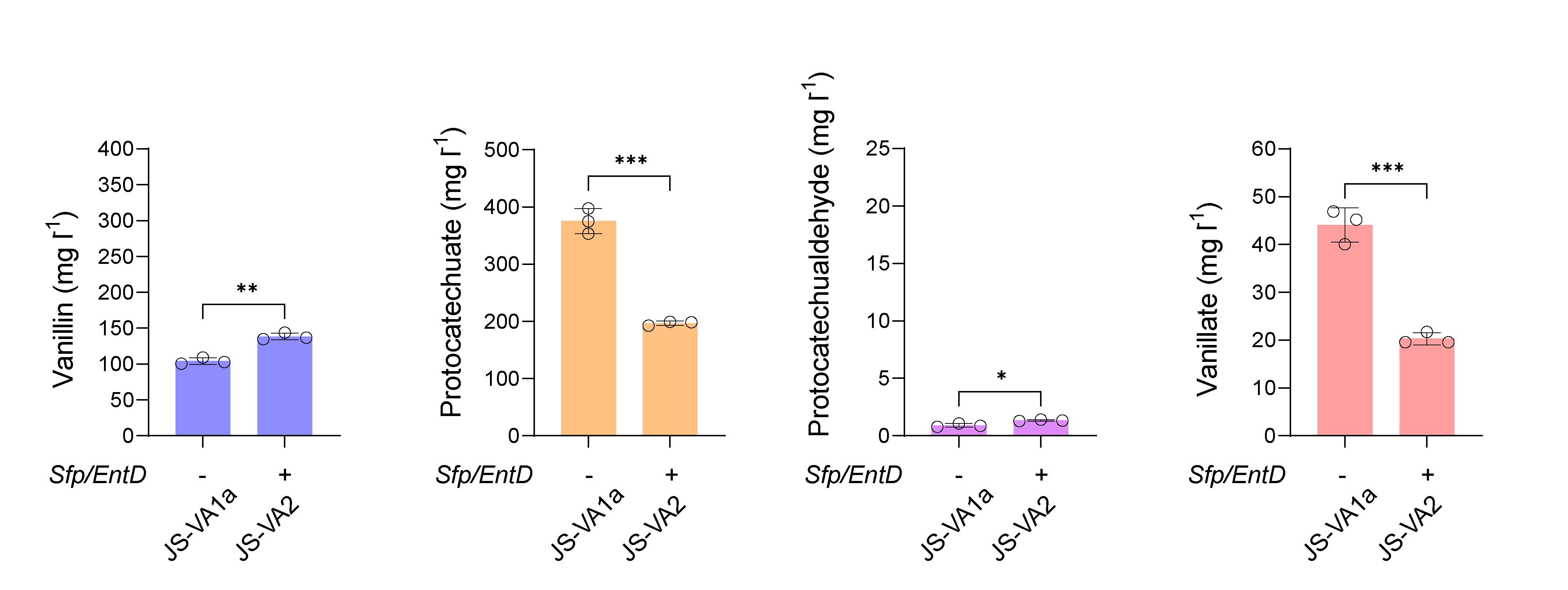


**Figure S4. The effect of Sfp and EntD expression on vanillin production.** The vanillin levels in strain JS-VA1a and JS-VA2 were compared. Cells were grown in SC medium with 2% glucose, and samples were measured after 120 h of cultivation. The experiments were performed in triplicate and the data represent the mean value with standard deviation.

**
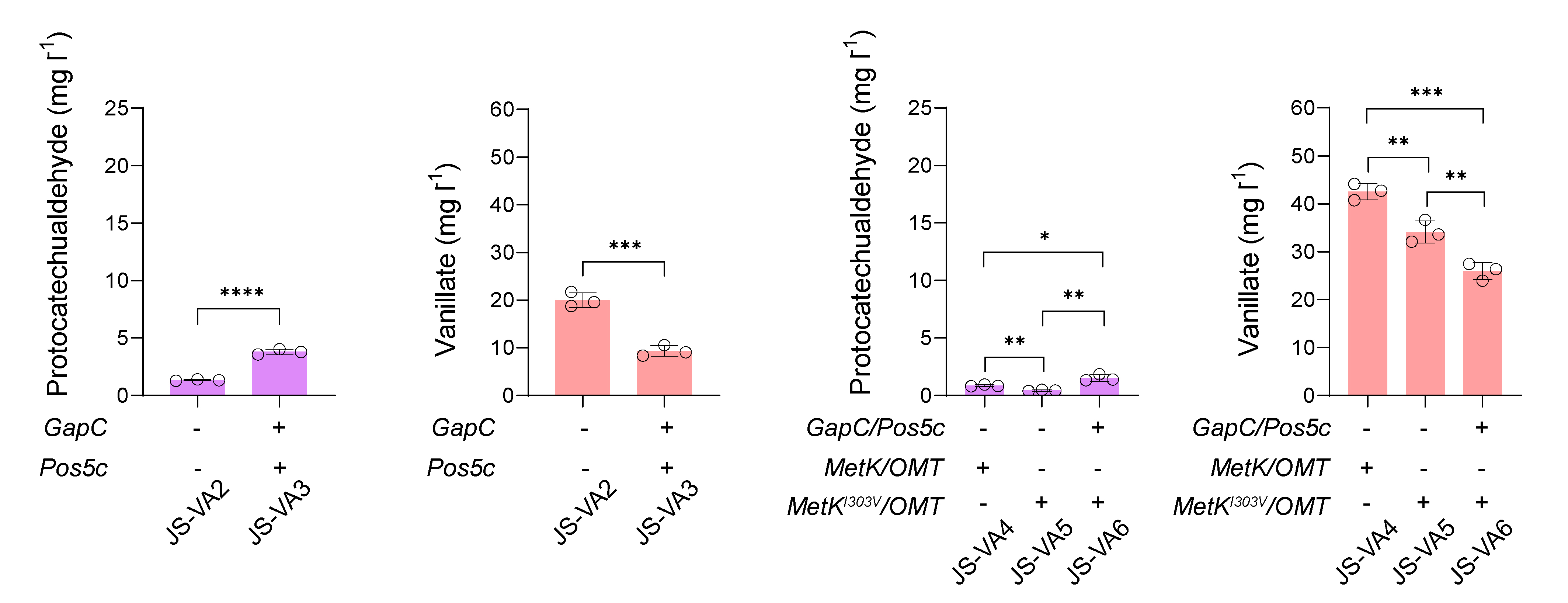
**

**Figure S5. Product profiles of protocatechualdehyde and vanillate produced by engineered yeasts of JS-VA2~6.** Cells were grown in SC medium with 2% glucose, and samples were measured after 120 h of cultivation. The experiments were performed in triplicate and the data represent the mean value with standard deviation.

**
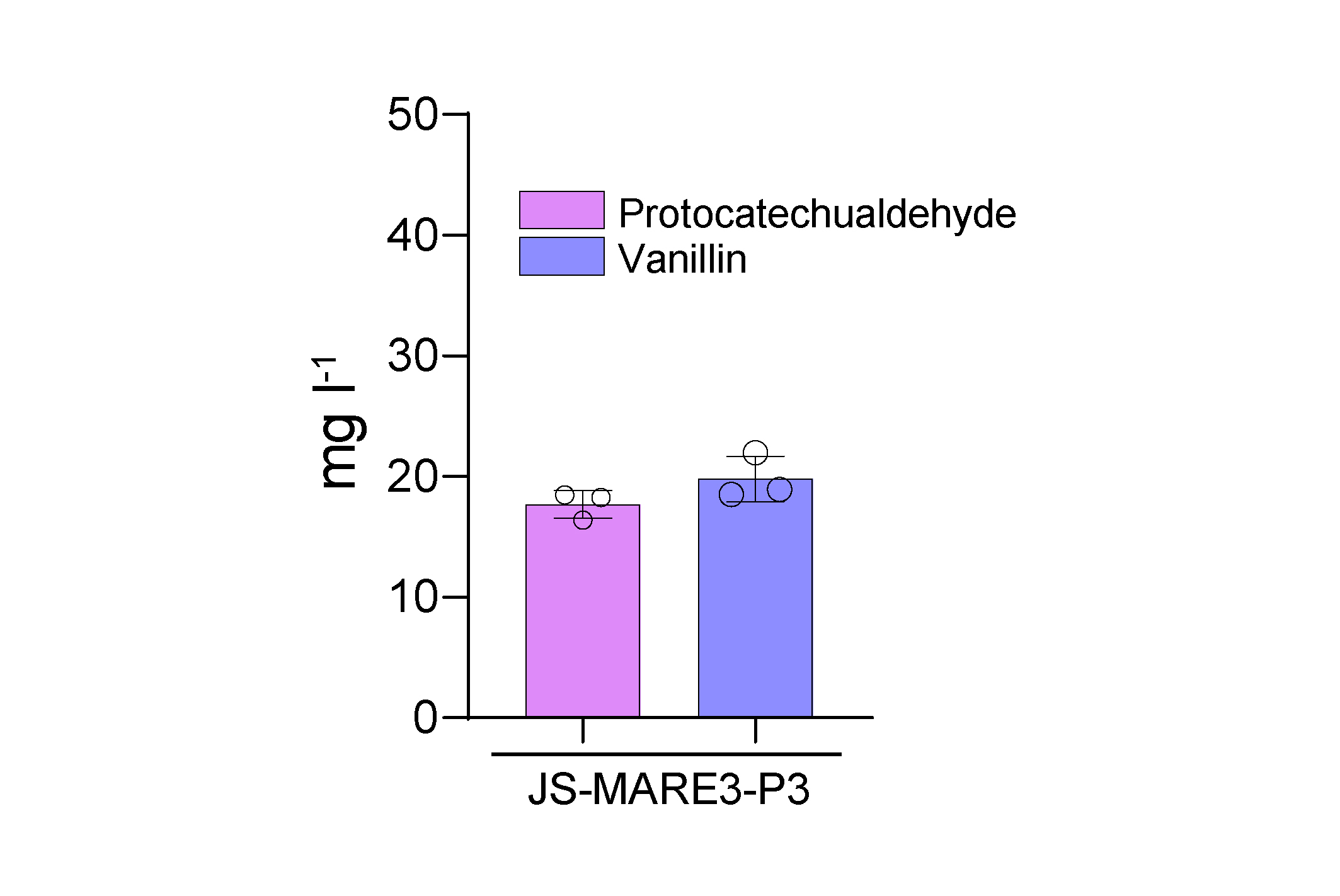
**

**Figure S6. *De novo* synthesis of vanillin from plasmid-based HmaS pathway in *S. cerevisiae*.** The strain JS-MARE3-P3 is a derivative from JS-MARE3 with plasmids pRS423-HpaB/HpaC, pRS425-BFD/HMO, and pRS426-HmaS/OMT. SC media supplemented with 2% (w/v) glucose with dropouts were used for cultivating the engineered yeasts. The experiments were performed in triplicate and the data represent the mean value with standard deviation.


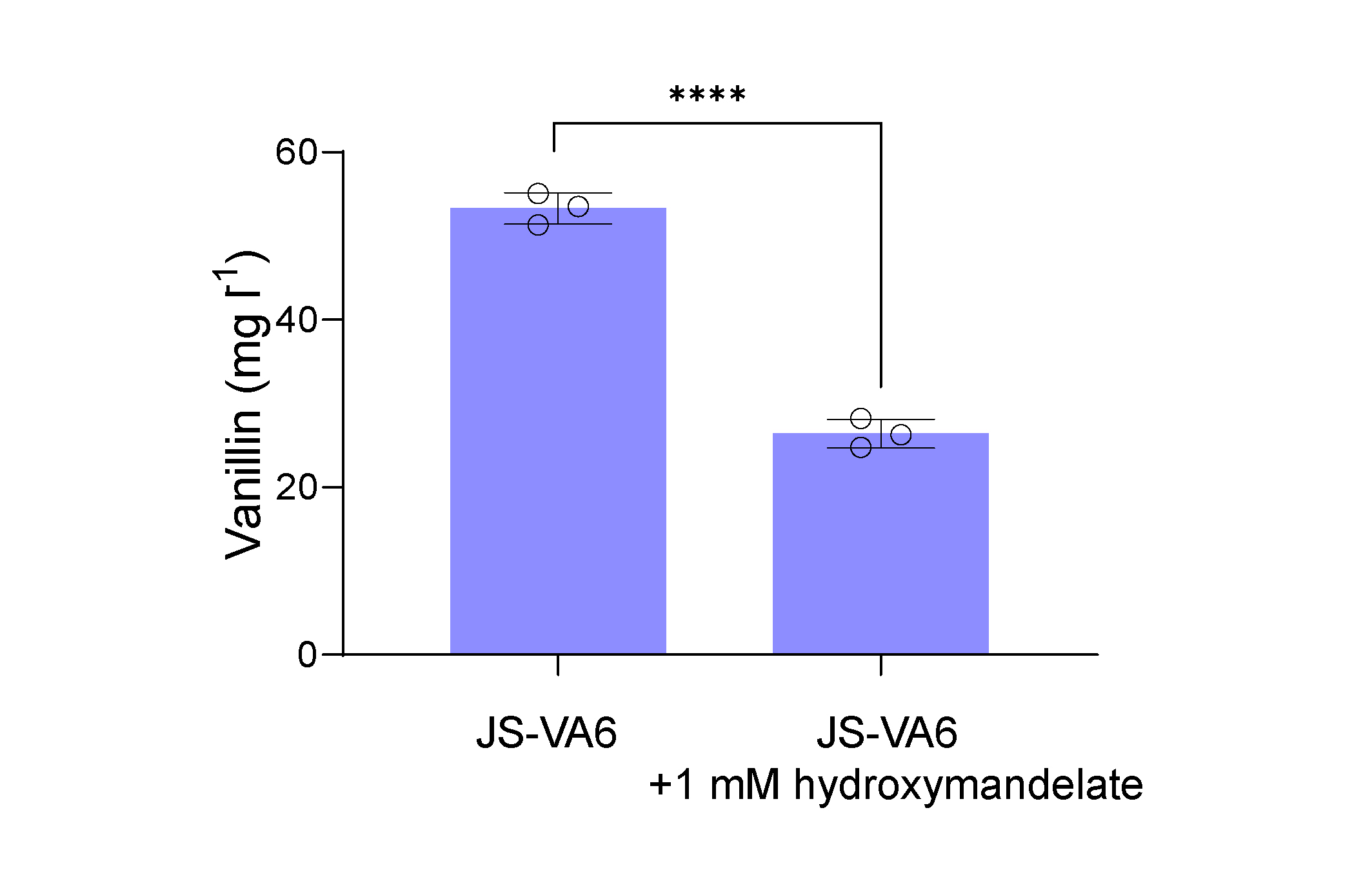


**Figure S7. The inhibitory effect of hydroxymandelate to the CAR-mediated vanillin biosynthetic pathway.** Comparison of the vanillin levels in *S. cerevisiae* strain (JS-VA6) with or without additional supplementation of 1 mM of hydroxymandelate. The vanillin levels in 14 ml shake tubes supplemented with 2 ml SC media were measured after 2 days. The experiments were performed in triplicate and the data represent the mean value with standard deviation.

**
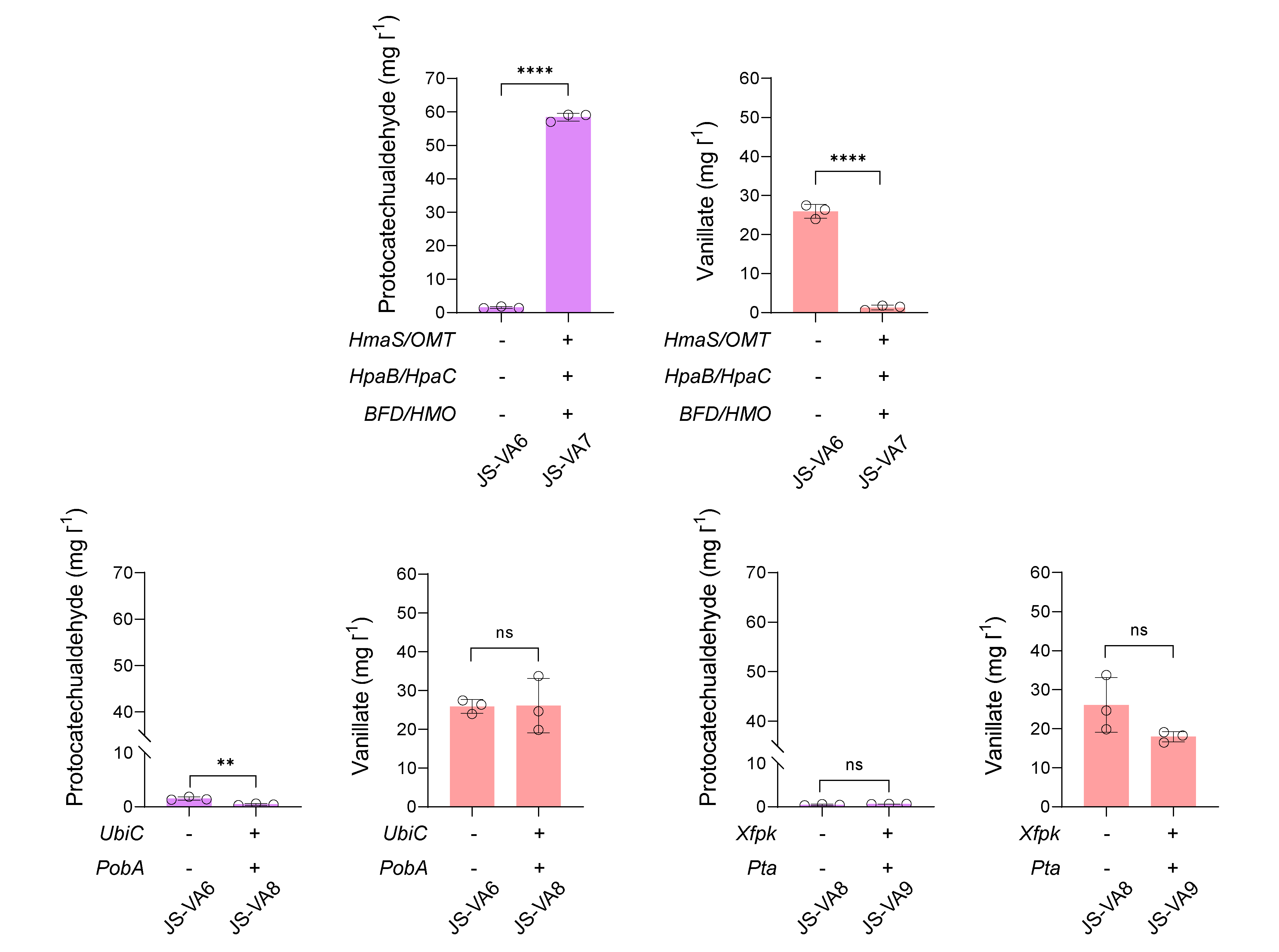
**

**Figure S8. Product profiles of protocatechualdehyde and vanillate produced by engineered yeasts of JS-VA6~9.** Cells were grown in SC medium with 2% glucose, and samples were measured after 120 h of cultivation. The experiments were performed in triplicate and the data represent the mean value with standard deviation.

**
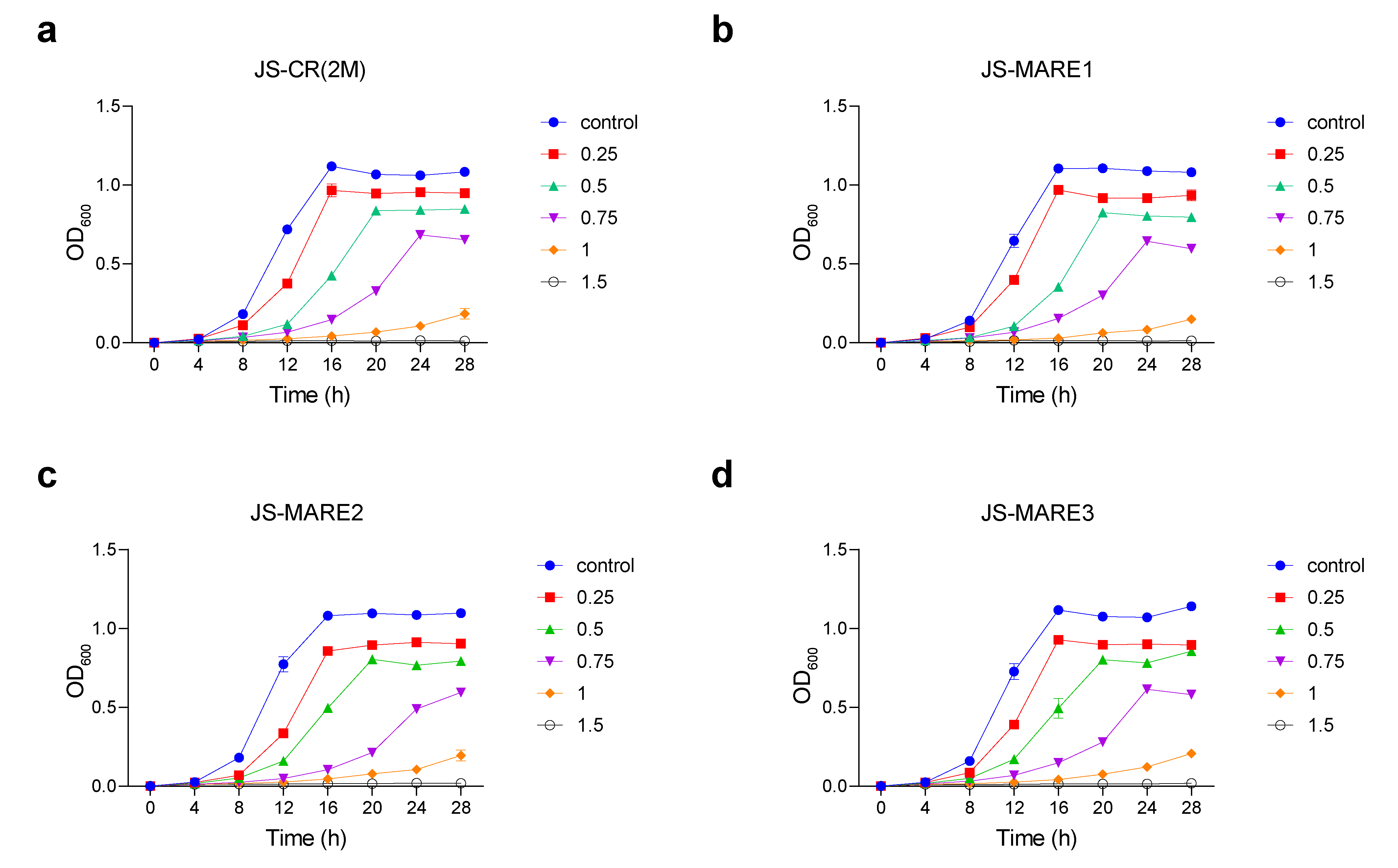
**

**Figure S9. The growth inhibitory effect of vanillin to *S. cerevisiae*.** Strain JS-CR(2M), JS-MARE1, JS-MARE2, and JS-MARE3 were treated with different concentrations of vanillin (blue dot, no vanillin supplementation; red square, 0.25 g/L vanillin; green triangle, 0.50 g/L vanillin; purple inverted triangle, 0.75 g/L vanillin; orange diamond, 1.0 g/L vanillin; black circle, 1.5 g/L vanillin).


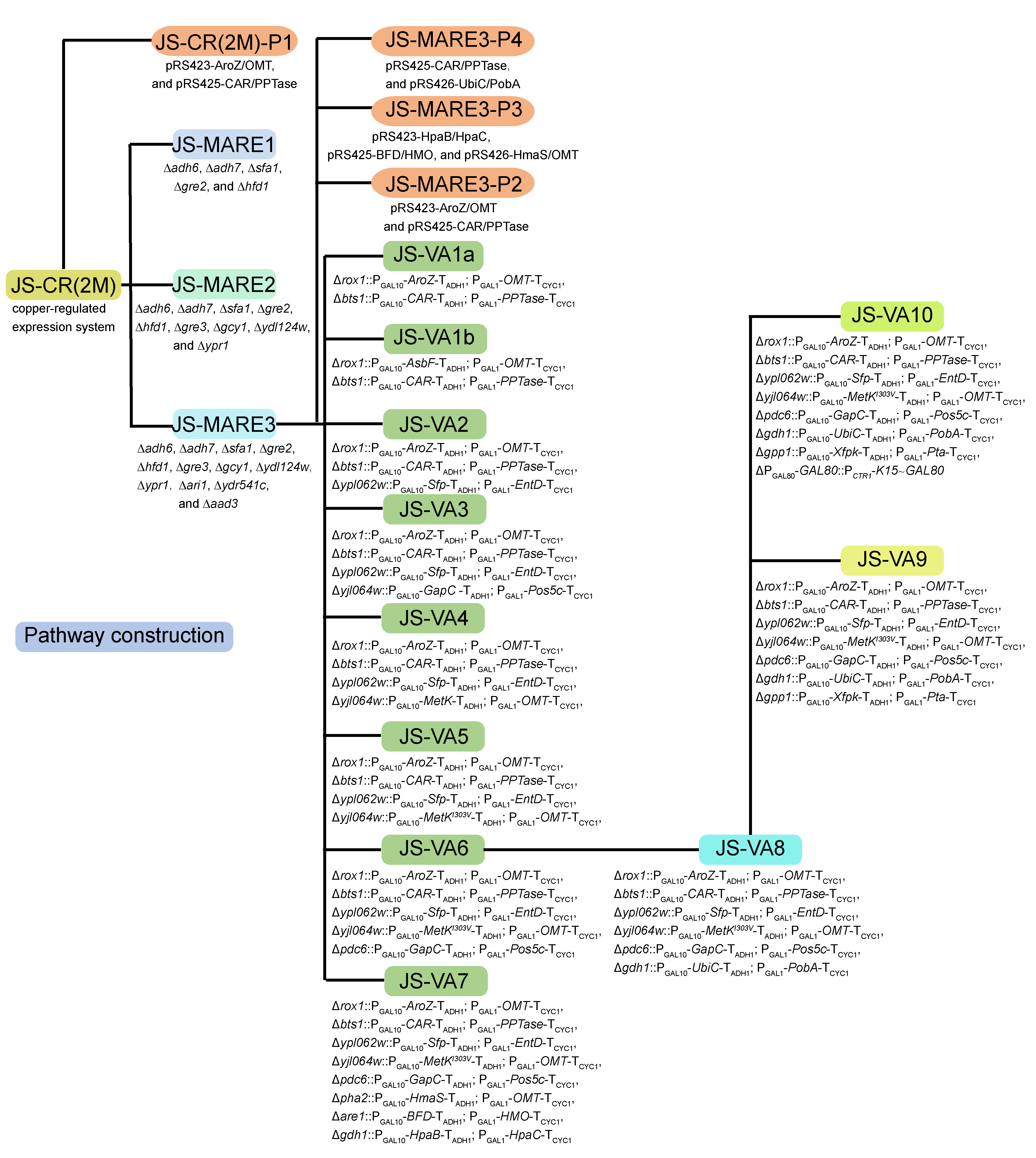


**Figure S10. Flowchart of yeast strain construction in this study.**

**Supplementary Tables**

**Table S1.** Oligonucleotides used in this study.

| **Name** | **Description** |
| --- | --- |
| F_gRNA.gre3 | TTGGTCTCAGATGATTCTCGAACAGAGTTGGCGGTTTTAGAGCTAGAAATAG |
| F_gRNA.gcy1 | TTGGTCTCAGATGTACCGATGCTCCACTATTGAGTTTTAGAGCTAGAAATAG |
| F_gRNA.ypr1 | TTGGTCTCAGATGCAAGTGCCGAAACCCAACACGTTTTAGAGCTAGAAATAG |
| F_gRNA.ydl124w | TTGGTCTCAGATGATGTCAGATTCCCCAGCGGAGTTTTAGAGCTAGAAATAG |
| F_gRNA.ari1 | TTGGTCTCAGATGAGGAGATTTGGTGATCACGGGTTTTAGAGCTAGAAATAG |
| F_gRNA.aad3 | TTGGTCTCAGATGGACTTCTCCTAGGATTAACGGTTTTAGAGCTAGAAATAG |
| F_gRNA.ydr541c | TTGGTCTCAGATGGGTGATCAAGTCATTGACCGGTTTTAGAGCTAGAAATAG |
| F_gRNA.pdc6 | TTGGTCTCAGATGGTTGGGGTCAATCTCCTCAGGTTTTAGAGCTAGAAATAG |
| F_gRNA.pha2 | TTGGTCTCAGATGTTGAAGAGAAGGGAGAACGCGTTTTAGAGCTAGAAATAG |
| F_gRNA.are1 | TTGGTCTCAGATGCTTCACCGTAGTCTTCACCGGTTTTAGAGCTAGAAATAG |
| F_gRNA.gdh1 | TTGGTCTCAGATGACAACACCCAGAATACAGAAGTTTTAGAGCTAGAAATAG |
| F_gRNA.gpp1 | TTGGTCTCAGATGACCGTCAACATCGAATAGAGGTTTTAGAGCTAGAAATAG |
| F_gRNA.gal80N | TTGGTCTCAGATGGGACTACAACAAGAGATCTTGTTTTAGAGCTAGAAATAG |
| R_SUP4 | TTGGTCTCAAAAGAGACATAAAAAACAAAAAAAG |
| F-adh6-Del | ATGTCTTATCCTGAGAAATTTGAAGGTATCGCTATTCAATTTACCTTAG |
| R-adh6-Del | CTAGTCTGAAAATTCTTTGTCGTAGCCGACTAAGGTAAATTGAATAGCG |
| F-adh7-Del | ATGCTTTACCCAGAAAAATTTCAGGGCATCGGTATTTCCTTTACTTTGG |
| R-adh7-Del | CTATTTATGGAATTTCTTATCATAATCGACCAAAGTAAAGGAAATACCG |
| F-sfa1-Del | ATGTCCGCCGCTACTGTTGGTAAACCTATTAAGTGCATTTGCTTAAGAA |
| R-sfa1-Del | CTATTTTATTTCATCAGACTTCAAGACGGTTCTTAAGCAAATGCACTTA |
| F-gre2-Del | ATGTCAGTTTTCGTTTCAGGTGCTAACGGGTTCATTGCCACTGCCTCCC |
| R-gre2-Del | TTATATTCTGCCCTCAAATTTTAAAATTTGGGAGGCAGTGGCAATGAAC |
| F-hfd1-Del | ATATTCTAAAACCATAGCCATAGTAATTTATCACCAACATGTCACACCCCGCGTTAAC |
| R-hfd1-Del | CTTATACATCAAATAATTAATTAACCTTAAACATTACGTTTAGTACAACGGTGACGCCG |
| F-gre3-Del | ATGTCTTCACTGGTTACTCTTAATAACGGTCTGAAAATGGAGCGACCTCATGCTATAC |
| R-gre3-Del | TCAGGCAAAAGTGGGGAATTTACCATCCAACCAGGTCCAGTATAGCATGAGGTCGCTC |
| F-ydl124w-Del | ATGTCATTTCACCAACAGTTCTTTACCTTGAATAATGGATTGTACGGTA |
| R-ydl124w-Del | TTATACTTTTTGAGCAGCGTAGTTGTATTTACCGTACAATCCATTATTC |
| F-gcy1-Del | ATGCCTGCTACTTTACATGATTCTACGAAAATCCTTTCTGAGCGACCTCATGCTATAC |
| R-gcy1-Del | TTACTTGAATACTTCGAAAGGAGACCAATTTGGATGTACGTATAGCATGAGGTCGCTC |
| F-ypr1-Del | ATGCCTGCTACGTTAAAGAATTCTTCTGCTACATTAAAAGAGCGACCTCATGCTATAC |
| R-ypr1-Del | TCATTGGAAAATTGGGAAGGATCCCCACTTCATATCAACGTATAGCATGAGGTCGCTC |
| F-ari1-Del | ATGACTACTGATACCACTGTTTTCGTTTCTGGCGCAACCAGCGACCTCATGCTATAC |
| R-ari1-Del | TTAGGCTTCATTTTGAACTTCTAACATTTGCGCCGCGGTGTATAGCATGAGGTCGCT |
| F-ydr541c-Del | ATGTCTAATACAGTTCTAGTTTCTGGCGCTTCAGGTTTTAGCGACCTCATGCTATAC |
| R-ydr541c-Del | TCATAATCTGTTCTGCTTCTTCAAAATTTGGGCAGCAGTGTATAGCATGAGGTCGCT |
| F-aad3-Del | ATGATTGGGTCCGCGTCCGACTCATCTAGCAAGTTAGGAAGCGACCTCATGCTATAC |
| R-aad3-Del | CTAAACATTATTCGTACCATATTTTTGAGTCAAGGAATGTATAGCATGAGGTCGCT |

| F-bts1-Int | ATGGAGGCCAAGATAGATGAGCTGATCAATAATGATCCTGGAGCGACCTCATGCTATAC |
| --- | --- |
| R-bts1-Int | TCACAATTCGGATAAGTGGTCTATTATATATAACAATTCGCTTCGAGCGTCCCAAAACC |
| F-ypl062w-Int | ATGATAGAATTGGATTATGTAAAAGGTGAAGATACCATTGGAGCGACCTCATGCTATAC |
| R-ypl062w-Int | CTATATCGCATTCGTTGCACTCACCGTTCCCAAGAGGAGACTTCGAGCGTCCCAAAACC |
| F-rox1-Int | ATGAATCCTAAATCCTCTACACCTAAGATTCCAAGACCCAGAGCGACCTCATGCTATAC |
| R-rox1-Int | TCATTTCGGAGAAACTAGGCTAGTTTTAGCGGTGACCTCACTTCGAGCGTCCCAAAACC |
| F-yjl064w-Int | ATGACACTTGTAGTATATCTAACTCGGTTTTCTTCCACTAGAGCGACCTCATGCTATAC |
| R-yjl064w-Int | TCAGGCTAACACAATGAACAACGAGACTAGTGGTAAAGAACTTCGAGCGTCCCAAAACC |
| F-pdc6-Int | ATGTCTGAAATTACTCTTGGAAAATACTTATTTGAAAGAGAGCGACCTCATGCTATAC |
| R-pdc6-Int | TTATTGTTTGGCATTTGTAGCGGCAGTCAATTGCGCTTGCTTCGAGCGTCCCAAAACC |
| F-pha2-Int | ATGGCCAGCAAGACTTTGAGGGTTCTTTTTCTGGGTCCCGAGCGACCTCATGCTATAC |
| R-pha2-Int | TTATTTGTGATAATATCTCTCATTTCTGGGGAATGTACCCTTCGAGCGTCCCAAAACC |
| F-are1-Int | ATGACGGAGACTAAGGATTTGTTGCAAGACGAAGAGTTTGAGCGACCTCATGCTATAC |
| R-are1-Int | TCATAAGGTCAGGTACAACGTCATAATGATACTGGGCCCCTTCGAGCGTCCCAAAACC |
| F-gdh1-Int | ATGTCAGAGCCAGAATTTCAACAAGCTTACGAAGAAGTTGAGCGACCTCATGCTATAC |
| R-gdh1-Int | TTAAAATACATCACCTTGGTCAAACATAGCATCAGAGACCTTCGAGCGTCCCAAAACC |
| F-gpp1-Int | ATGCCTTTGACCACAAAACCTTTATCTTTGAAAATCAACGAGCGACCTCATGCTATAC |
| R-gpp1-Int | TTACCATTTCAACAAGTCATCCTTAGCGTATAAGTAGTCCTTCGAGCGTCCCAAAACC |
| Ubi-K15N_int_fwd | TAGAAAATAAAAAAAAGTGTATTATATTTGACATTCAAAATGCAGATTTTCGTCAAGAC |
| Ubi-K15N_int_rev | CCGACTCTTATGGGAGCTGCATTAGGCACGGTTGAGACACCAGAACCCTTAACCAAAG |
| F-adh6v | ACAGCCACTCTCGTCACGGC |
| R-adh6v | CCGATACCCTATGAACGTGC |
| F-adh7v | GATACGTTTGGCTCTGTTGC |
| R-adh7v | CACTGTTGTCGAGAGATTC |
| F-sfa1v | AGACATGCGGTGTGTGGGTC |
| R-sfa1v | GTTAGGAACAGGCGAGGTC |
| F-gre2v | ACATTGTTGTACGCTATAG |
| R-gre2v | GCTTATCTGAACGTTTCTC |
| F-hfd1v | CTTAGAGGAAATGGAACAAC |
| R-hfd1v | GAAAGGTTACTTATACATC |
| F-gre3v | CGCAGATACTGTAAATGCCG |
| R-gre3v | ATGGCTAGTGCTATCATTGC |
| F-ydl124wv | CTGTAACGATTCGCACCATATC |
| R-ydl124wv | GCGAGTTTTGAGGACGATTC |
| F-gcy1v | ATGCCTGCTACTTTACATG |
| R-gcy1v | CTTCTGGTGGCCTATCTTTG |
| F-ypr1v | CCATGCCTGCTACGTTAAAG |
| R-ypr1v | ATGAAGGAGAAGAAGATTCG |
| F-ari1v | ACCTTAGACCTTGCAACATC |
| R-ari1v | TTAGGCTTCATTTTGAACTTC |
| F-ydr541cv | CAGCAACAATGGCCAGTCGC |
| R-ydr541cv | TCATAATCTGTTCTGCTTCTTC |
| F-aad3v | CAATATCAGCGAAGAAGAGG |

| R-aad3v | CTCTACCACCTTCGTCAGAG |
| --- | --- |
| M1_bts1_F300 | CGCCATCTCTACTCACTCC |
| M1_bts1_R300 | TGTCTAGGGATGGCTCACG |
| M2_ypl062w_F300 | CACACCTAATGATCTGATGC |
| M2_ypl062w_R300 | AGTGTGATCTTGACTGGTTC |
| M3_rox1_F300 | CTCGTATTGTCTTGCCGGTG |
| M3_rox1_R300 | TATCGATAATATAGGTATAC |
| M4_yjl064w_F200 | CGTATAAAGAAACTATGTTG |
| M4_yjl064w_R200 | CTATGTCATGGTTCCCAGAC |
| Pdc6_F800 | CTTTCCTCACGGACCATACG |
| Pdc6_R0 | GTTTGGCATTTGTAGCGGC |
| Pha2_F200 | CGTACTACATCATCTGCGAC |
| Pha2_R200 | CGGTGCGGCCCCGCCACCAC |
| Are1_F200 | CTGGCTTGGCCATCAAATACC |
| Are1_R200 | GTCACCTGCAAACTCTTCTTG |
| Gdh1_F200 | GTATACGTAATCTAAGTAAG |
| Gdh1_R200 | GTCCAATCGATGCTTACATAC |
| Gpp1_F100 | TCTTTCGTAAGTATCTCTTG |
| Gpp1_R150 | GAAAAGGATGCATTACATCG |
| AroZ_P1_fwd | TTGGTCTCATGAAAACAATGCCAAGTAAACTGGCTAT |
| AroZ_P1_rev | TTGGTCTCAATCTATTAAAGTGCGGCAGATAGAG |
| OMT_P2_fwd | TTGGTCTCAAACCAATGGGCGATACCAAAGAAC |
| OMT_P2_rev | TTGGTCTCATCTTACGGACCTGCTTCGCTAC |
| CAR_P1_fwd | TTGGTCTCATGAAAACAATGACCCAGTCTCACACCC |
| CAR_P1_rev | TTGGTCTCAATCTACAGCAGACCCAGCTGTTTG |
| PPTase_P2_fwd | TTGGTCTCAAACCAATGATCGAAACCATCCTGC |
| PPTase_P2_rev | TTGGTCTCATCTTAAGCGTAAGCGATCGCGG |
| Sfp_P1_fwd | TTGGTCTCATGAAACAATGAAGATTTACGGAATTTATATG |
| Sfp_P1_rev | TTGGTCTCAATCTATAAAAGCTCTTCGTACGAAACCATTG |
| EntD_P2_fwd | TTGGTCTCAAACCAATGGTCGATATGAAAACTAC |
| EntD_P2_rev | TTGGTCTCATCTTAATCGTGTTGGCACAGCG |
| GapC_P1_fwd | TTGGTCTCATGAAAACAATGGCAAAGATAGCTATTAATG |
| GapC_P1_rev | TTGGTCTCAATCTATTTTGCTATTTTTGCAAAGTAAG |
| Pos5c_P2_fwd | TTGGTCTCAAACCAATGAGTACGTTGGATTCACATTCC |
| Pos5c_P2_rev | TTGGTCTCATCTTAATCATTATCAGTCTGTCTC |
| MetK_P1_fwd | TTGGTCTCATGAAAACAATGGCAAAACACCTTTTTACG |
| MetK_OE_rev | CAGCCACGCCTACTGCGTAGGAAACC |
| MetK_OE_fwd | CCTACGCAGTAGGCGTGGCTGAACCG |
| MetK_P1_rev | TTGGTCTCAATCTACTTCAGACCGGCAGCATC |
| HmaS_P1_fwd | TTGGTCTCATGAAAACAATGCAGAATTTCGAAATCG |
| HmaS_P1_rev | TTGGTCTCAATCTAACGCCTCGCGGCTCCAAAC |
| BFD_P1_fwd | TTGGTCTCATGAAAACAATGGCTTCGGTACACGGC |
| BFD_OE_rev | GTCGAACACAGTCTCCGGGTG |

| BFD_OE_fwd | CACCCGGAGACTGTGTTCGAC |
| --- | --- |
| BFD_P1_rev | TTGGTCTCAATCTACTTCACCGGGCTTACGG |
| HMO_P2_fwd | TTGGTCTCAAACCAATGCGTGAACCGCTGACGC |
| HMO_P2_rev | TTGGTCTCATCTTAGCCGTGAGAACGATCGC |
| HpaB_P1_fwd | TTGGTCTCATGAAAACAATGAAACCCGAAGATTTCCG |
| HpaB_P1_rev | TTGGTCTCAATCTATTGGCGGATGCGATCGAGCAC |
| HpaC_P2_fwd | TTGGTCTCAAACCAATGCAAGTAGATGAACAACG |
| HpaC_P2_rev | TTGGTCTCATCTTAAACAGGCGCTTCCATCTC |
| UbiC_P1_fwd | TTGGTCTCATGAAAACAATGGTCACACCCCGCGTTAAC |
| UbiC_P1_rev | TTGGTCTCAATCTAGGTACAACGGTGACGCCGG |
| PobA_P2_fwd | TTGGTCTCAAACCAATGAAAACTCAGGTTGCAAT |
| PobA_P2_rev | TTGGTCTCATCTTAGGCAACTTCCTCGAACGGC |
| Xfpk_P1_fwd | TTGGTCTCATGAAAACAATGACTTCTCCAGTTATC |
| Xfpk_P1_rev | TTGGTCTCAATCTATTCGTTATCACCAGCAGTTG |
| Pta_P2_fwd | TTGGTCTCAAACCAATGAAGTTGATGGAAAACATC |
| Pta_P2_rev | TTGGTCTCATCTTAACCTTGAGCTTGGGCTTG |

**Table S2** Synthesized genes used in this study.

| **Name** | **Description** |
| --- | --- |
| *OMT* from *H. sapiens* | ATGGGCGATACCAAAGAACAGCGTATTCTGAATCATGTTCTGCAGCATGCCGAACCGGGTAATGCACAGAGCGTTCTGGAAGCAATTGATACCTATTGTGAACAGAAAGAATGGGCCATGAATGTGGGTGATAAAAAAGGCAAAATTGTGGATGCCGTGATCCAAGAACATCAGCCGAGCGTGCTGCTGGAACTGGGTGCATATTGTGGTTATAGCGCAGTTCGTATGGCACGTCTGCTGAGTCCGGGTGCACGTCTGATTACCATTGAAATTAACCCGGATTGTGCAGCAATTACCCAGCGTATGGTTGATTTTGCCGGTGTTAAAGATAAAGTTACCCTGGTTGTTGGTGCAAGCCAGGATATTATTCCGCAGCTGAAAAAAAAATATGACGTGGATACCCTGGATATGGTGTTTCTGGATCATTGGAAAGATCGTTATCTGCCGGATACCCTGCTGCTGGAAGAATGTGGTCTGCTGCGTAAAGGCACCGTTCTGCTGGCAGATAATGTTATTTGTCCTGGTGCACCGGATTTTCTGGCACATGTTCGTGGTAGCAGCTGTTTTGAATGTACCCATTATCAGTCCTTTCTGGAATATCGTGAAGTTGTTGATGGTCTGGAAAAAGCCATCTATAAAGGTCCGGGTAGCGAAGCAGGTCCGTAA |
| *HmaS* from *A. orientalis* | ATGCAGAATTTCGAAATCGACTATGTTGAGATGTATGTGGAGAATCTGGAAGTGGCGGCTTTCTCATGGGTCGACAAGTACGCATTCGCCGTGGCTGGTACGAGCCGTTCAGCGGACCACAGGAGCATCGCGTTGAGACAAGGGCAGGTGACACTTGTTCTTACTGAACCGACGTCAGATAGACATCCGGCCGCCGCGTATTTGCAGACTCATGGCGATGGTGTAGCGGATATTGCTATGGCCACCTCCGACGTTGCTGCGGCCTACGAGGCGGCAGTACGTGCCGGTGCTGAAGCTGTTAGAGCACCAGGGCAACACAGTGAAGCTGCTGTGACGACCGCGACCATAGGTGGGTTTGGAGATGTCGTCCATACTCTGATCCAAAGGGACGGCACTAGCGCTGAATTACCCCCCGGATTTACCGGCTCCATGGACGTTACGAACCATGGTAAAGGAGACGTAGATCTTCTGGGGATAGATCACTTTGCGATATGTCTTAACGCCGGAGATCTGGGACCTACTGTGGAATACTACGAGAGAGCTTTAGGTTTTAGGCAAATATTTGACGAGCATATAGTTGTTGGCGCTCAGGCGATGAACTCAACTGTCGTGCAAAGTGCGAGTGGGGCGGTAACACTAACCTTGATAGAACCCGATCGTAATGCCGACCCCGGGCAAATTGATGAGTTCCTAAAAGATCACCAGGGTGCGGGTGTGCAGCACATCGCCTTTAATTCTAATGACGCTGTGCGTGCAGTGAAAGCACTGTCAGAGAGAGGGGTCGAGTTTTTGAAAACCCCGGGGGCGTATTACGATCTTCTAGGAGAGAGAATAACCCTGCAAACGCATAGTCTGGATGATTTGAGAGCGACCAATGTTTTGGCAGATGAGGATCACGGAGGTCAACTTTTTCAAATCTTCACAGCGAGTACGCACCCAAGACACACCATTTTTTTTGAAGTCATCGAAAGACAAGGAGCGGGCACTTTTGGTTCCAGTAATATCAAGGCTTTATATGAGGCAGTGGAACTAGAACGTACAGGTCAAAGTGAGTTTGGAGCCGCGAGGCGTTAG |

| *HpaB* from *P. aeruginosa* | ATGAAACCCGAAGATTTCCGAGCTTCTGCTACCAGACCATTCACCGGTGAAGAATACTTAGCCAGCCTTCGTGATGATCGTGAAATCTACATCTATGGTGACCGTGTCAAGGACGTTACTTCTCATCCAGCTTTCAGAAACGCTGCTGCTTCTATGGCTAGATTGTACGATGCCTTGCACGACCCACAATCGAAGGAAAAGTTGTGTTGGGAAACTGACACTGGTAACGGTGGTTACACTCACAAGTTCTTCAGATACGCCAGATCCGCTGACGAATTGAGACAACAAAGAGATGCCATCGCTGAATGGTCCAGATTGACCTACGGTTGGATGGGTAGAACCCCAGACTACAAGGCTGCTTTTGGTTCTGCATTAGGTGCTAACCCAGGTTTCTACGGTCGTTTTGAAGACAATGCCAAGACCTGGTATAAGAGAATCCAAGAAGCCTGTTTGTACTTGAACCACGCTATCGTCAACCCACCAATTGACCGTGACAAGCCAGTTGACCAAGTTAAGGATGTTTTCATCTCTGTCGATGAAGAAGTCGATGGTGGTATTGTCGTCAGTGGTGCTAAGGTTGTTGCTACCAACTCCGCTCTAACCCACTACAACTTCGTTGGTCAAGGTTCTGCTCAATTGTTGGGTGACAACACCGATTTCGCTTTGATGTTCATTGCTCCAATGAACACTCCAGGTATGAAGTTGATCTGTCGGCCTTCTTACGAATTGGTCGCTGGTATCGCCGGTTCTCCATTTGACTACCCTTTGTCTTCTAGATTCGATGAAAACGACGCTATTTTGGTTATGGACAAGGTGTTCATTCCTTGGGAAAACGTTTTGATTTACAGAGACTTCGAAAGATGTAAGCAATGGTTTCCACAAGGTGGTTTCGGCAGATTATTCCCAATGCAAGGTTGTACTAGATTAGCTGTCAAATTGGACTTCATCACTGGTGCCCTTTACAAGGCTTTGCAATGCACCGGTTCCTTGGAATTTCGTGGTGTCCAAGCTCAAGTCGGTGAAGTTGTTGCTTGGAGAAACTTGTTCTGGTCTTTGACAGATGCTATGTACGGTAACGCGTCTGAATGGCACGGTGGTGCTTTCTTGCCATCTGCCGAGGCTTTGCAAGCCTACAGAGTTTTGGCTCCACAAGCCTACCCAGAAATCAAGAAGACTATTGAACAAGTTGTCGCTTCTGGTTTGATTTACTTGCCATCCGGTGTTAGAGACTTACACAACCCTCAACTGGACAAGTACCTATCCACTTACTGTAGAGGTTCTGGAGGTATGGGTCACAGAGAAAGAATCAAGATTTTGAAGCTATTGTGGGATGCTATCGGGTCAGAATTTGGTGGTAGACACGAATTATACGAAATCAACTACGCCGGTTCCCAGGATGAAATAAGAATGCAAGCCTTGAGACAAGCCATTGGTTCCGGTGCCATGAAGGGTATGTTGGGTATGGTTGAACAATGTATGGGTGATTATGACGAAAATGGTTGGACTGTTCCACATTTGCATAACCCAGATGACATTAACGTGCTCGATCGCATCCGCCAATGA |
| --- | --- |

| *Xfpk* from *B. breve* | ATGACTTCTCCAGTTATCGGTACTCCTTGGAAGAAGTTGAACGCTCCTGTTTCAGAAGAATCTTTGGAAGGTGTTGACAAGTACTGGAGAGTTGCTAACTACTTGTCCATCGGTCAAATCTACTTAAGATCCAACCCATTGATGAAGGCCCCATTCACTAGAGAAGATGTTAAGCACCGTTTGGTTGGTCACTGGGGTACTACCCCAGGTTTGAACTTCTTGATTGGTCACATCAACAGATTCATCGCTGACCACGGTCAAAACACCGTCATCATTATGGGTCCAGGTCACGGTGGACCGGCTGGTACTTCTCAGTCCTATTTGGATGGTACTTACACCGAAACTTTCCCAAAAATCACCAAGGACGAAGCTGGCTTGCAAAAGTTTTTCAGACAATTCTCCTACCCAGGTGGTATTCCATCTCATTTCGCTCCAGAAACTCCTGGTTCTATCCACGAAGGTGGTGAATTGGGTTACGCCTTGTCCCACGCGTACGGTGCTATCATGGACAACCCCTCTTTATTCGTTCCAGCTATTGTCGGTGATGGCGAAGCCGAAACTGGTCCATTGGCTACCGGTTGGCAATCTAACAAATTGGTGAACCCACGTACCGATGGTATTGTTTTGCCAATTTTGCATTTGAACGGTTATAAGATAGCTAACCCAACCATTCTGTCTAGAATTTCCGATGAAGAGTTGCACGAATTTTTCCACGGTATGGGTTACGAACCATACGAATTTGTTGCTGGTTTCGACGATGAAGATCACATGTCTATTCACAGAAGATTTGCTGAATTGTGGGAAACCATCTGGGACGAAATTTGCGACATCAAGGCGGCTGCTCAAACTGACAACGTCCACAGACCATTCTACCCAATGTTGATCTTCAGAACCCCAAAGGGTTGGACTTGTCCAAAGTACATTGACGGCAAGAAGACTGAAGGTTCTTGGAGAGCCCACCAAGTTCCACTCGCTTCGGCTAGAGACACTGAAGCCCACTTCGAAGTTCTGAAGAACTGGTTAGAATCCTACAAGCCAGAAGAATTATTTGATGCCAACGGGGCAGTCAAGGATGACGTCTTGGCCTTCATGCCAAAGGGTGAATTGAGAATTGGTGCTAACCCAAATGCTAATGGTGGTGTTATTAGGGATGATTTGAAGCTGCCAAACTTGGAAGACTACGAAGTCAAGGAAGTTGCTGAATACGGTCACGGCTGGGGTCAATTGGAAGCCACCCGTACGCTAGGTGCTTATACCCGGGATATAATCAGAAACAACCCTAGAGATTTCAGAATTTTCGGTCCAGACGAAACTGCCTCCAACAGATTGCAAGCCTCTTACGAAGTAACAAACAAGCAATGGGATGCTGGTTACATCTCTGACGAAGTCGATGAACACATGCATGTTTCCGGTCAAGTTGTTGAACAATTGTCTGAACATCAAATGGAAGGTTTTTTGGAAGCCTACTTGCTAACTGGTAGACACGGTATCTGGTCTTCTTACGAATCTTTCGTTCATGTCATCGACTCCATGTTGAACCAACACGCTAAGTGGTTGGAAGCTACTGTCAGAGAAATCCCTTGGAGAAAGCCAATCGCTTCGATGAACTTGTTGGTTTCTTCTCACGTTTGGCGTCAAGATCATAACGGTTTCTCTCACCAAGACCCAGGTGTTACTAGCGTTCTATTGAATAAGTGTTTCCACAACGATCACGTTATCGGTATTTACTTCGCCACCGACGCTAACATGTTGTTGGCTATCGCTGAAAAGTGTTACAAGTCTACCAACAAGATCAACGCCATTATTGCCGGTAAGCAACCAGCCGCTACCTGGTTAACTTTGGACGAAGCCAGAGCTGAACTGGCCAAGGGTGCTGCAGCTTGGGACTGGGCTTCCACTGCCAAGAACAATGATGAAGCTGAGGTCGTCTTGGCTGCCGCTGGTGACGTCCCAACTCAAGAAATTATGGCTGCGTCTGACAAGTTGAAGGAATTAGGTGTCAAATTCAAGGTTGTTAACGTTGCTGACTTGTTATCTTTGCAAAGTGCTAAGGAAAACGATGAAGCCTTGTCTGACGAAGAATTTGCTGATATTTTCACTGCTGACAAACCAGTCTTGTTCGCTTACCACTCTTACGCTCACGACGTTAGAGGTTTGATTTACGACAGACCAAACCATGATAACTTCAACGTTCACGGTTACGAAGAAGAAGGTTCCACCACCACCCCATACGACATGGTTAGAGTCAATAGAATCGATAGATACGAATTAACCGCTGAGGCTTTGAGAATGATCGATGCTGATAAGTACGCTGATAAGATAGACGAATTAGAAAAGTTCCGTGACGAAGCCTTCCAATTTGCTGTTGACAAAGGTTACGACCACCCAGATTACACAGACTGGGTTTACTCTGGTGTTAACACTGACAAGAAAGGTGCTGTCACTGCCACTGCCGCAACTGCTGGTGATAACGAATAG |
| --- | --- |
| *Pta* from *C. kluyveri* | ATGAAGTTGATGGAAAACATCTTCGGACTGGCTAAGGCTGACAAGAAGAAGATTGTCTTAGCTGAAGGTGAAGAAGAGCGTAACATTCGTGCTTCCGAAGAAATCATCAGAGATGGTATTGCTGACATTATCTTGGTCGGTTCTGAATCCGTTATCAAGGAAAACGCCGCTAAGTTCGGTGTTAACTTGGCTGGTGTCGAAATTGTTGACCCAGAAACTTCTTCTAAGACCGCCGGTTATGCTAACGCTTTCTACGAAATTAGAAAGAACAAGGGTGTTACTTTGGAAAAGGCTGATAAGATCGTTAGAGATCCTATCTACTTCGCTACCATGATGGTTAAGTTGGGTGACGCTGATGGTTTGGTTTCCGGTGCCATTCACACTACTGGTGATTTGTTAAGACCAGGCTTGCAAATCGTCAAGACTGTCCCAGGTGCTAGTGTTGTCTCTTCCGTTTTCTTAATGTCTGTTCCAGATTGTGAATACGGTGAAGATGGTTTCTTGTTATTTGCCGACTGTGCTGTCAACGTCTGTCCAACTGCTGAAGAATTATCTTCAATTGCCATCACCACTGCCGAAACCGCCAAGAACTTGTGTAAGATTGAACCAAGAGTTGCTATGTTGTCTTTCTCTACTATGGGTTCTGCTTCCCACGAATTGGTTGACAAAGTTACCAAGGCCACCAAGTTGGCTAAAGAAGCTAGACCAGACTTGGACATTGACGGTGAACTACAATTGGATGCTTCTTTGGTCAAAAAAGTGGCAGACTTGAAGGCTCCAGGTTCCAAGGTTGCTGGTAAGGCTAATGTTTTGATTTTCCCAGACATCCAAGCTGGTAACATTGGTTACAAGCTAGTTCAAAGATTCGCCAAAGCTGAAGCTATCGGTCCAATCTGCCAAGGTTTTGCTAAGCCAATCAACGATTTGAGCAGAGGTTGTTCTGTCGATGACATCGTCAAGGTTGTTGCTGTCACCGCCGTCCAAGCCCAAGCTCAAGGTTAA |

**Table S3** Plasmids used in this study.

| **Name** | **Description** |
| --- | --- |
| p414-TEF1-Cas9 ^1^ | Plasmid harboring *Cas9* gene under the control of TEF1 promoter with TRP selection marker |
| pRS426SNR52 | Plasmid harboring P_SNR52_-T_SUP4_ cassette, lab stock |
| pRS426-gRNA(adh6) ^2^ | pRS426SNR52 derivative with P_SNR52_-gRNA_adh6-T_SUP4_ |
| pRS426-gRNA(adh7) ^2^ | pRS426SNR52 derivative with P_SNR52_-gRNA_adh7-T_SUP4_ |
| pRS426-gRNA(sfa1) ^2^ | pRS426SNR52 derivative with P_SNR52_-gRNA_sfa1-T_SUP4_ |
| pRS426-gRNA(gre2) ^2^ | pRS426SNR52 derivative with P_SNR52_-gRNA_gre2-T_SUP4_ |
| pRS426-gRNA(hfd1) ^2^ | pRS426SNR52 derivative with P_SNR52_-gRNA_hfd1-T_SUP4_ |
| pRS426-gRNA(gre3) | pRS426SNR52 derivative with P_SNR52_-gRNA_gre3-T_SUP4_ |
| pRS426-gRNA(ydl124w) | pRS426SNR52 derivative with P_SNR52_-gRNA_ydl124w-T_SUP4_ |
| pRS426-gRNA(gcy1) | pRS426SNR52 derivative with P_SNR52_-gRNA_gcy1-T_SUP4_ |
| pRS426-gRNA(ypr1) | pRS426SNR52 derivative with P_SNR52_-gRNA_ypr1-T_SUP4_ |
| pRS426-gRNA(ari1) | pRS426SNR52 derivative with P_SNR52_-gRNA_ari1-T_SUP4_ |
| pRS426-gRNA(ydr541c) | pRS426SNR52 derivative with P_SNR52_-gRNA_ydr541c-T_SUP4_ |
| pRS426-gRNA(aad3) | pRS426SNR52 derivative with P_SNR52_-gRNA_aad3-T_SUP4_ |
| pRS426-gRNA(bts1) ^2^ | pRS426SNR52 derivative with P_SNR52_-gRNA_bts1-T_SUP4_ |
| pRS426-gRNA(ypl062w) ^2^ | pRS426SNR52 derivative with P_SNR52_-gRNA_ypl062w-T_SUP4_ |
| pRS426-gRNA(rox1) ^2^ | pRS426SNR52 derivative with P_SNR52_-gRNA_rox1-T_SUP4_ |
| pRS426-gRNA(yjl064w) ^2^ | pRS426SNR52 derivative with P_SNR52_-gRNA_yjl064w-T_SUP4_ |
| pRS426-gRNA(pdc6) | pRS426SNR52 derivative with P_SNR52_-gRNA_pdc6-T_SUP4_ |
| pRS426-gRNA(pha2) | pRS426SNR52 derivative with P_SNR52_-gRNA_pha2-T_SUP4_ |
| pRS426-gRNA(are1) | pRS426SNR52 derivative with P_SNR52_-gRNA_are1-T_SUP4_ |
| pRS426-gRNA(gdh1) | pRS426SNR52 derivative with P_SNR52_-gRNA_gdh1-T_SUP4_ |
| pRS426-gRNA(gpp1) | pRS426SNR52 derivative with P_SNR52_-gRNA_gpp1-T_SUP4_ |
| pRS426-gRNA(gal80N) | pRS426SNR52 derivative with P_SNR52_-gRNA_gal80N-T_SUP4_ |
| pRS423-AsbF/OMT | pRS423A-GGA derivative with P_GAL10_-*AsbF*-T_ADH1_; P_GAL1_-*OMT*-T_CYC1_ |
| pRS423-AroZ/OMT | pRS423A-GGA derivative with P_GAL10_-*AroZ*-T_ADH1_; P_GAL1_-*OMT*-T_CYC1_ |
| pRS425-Sfp/EntD | pRS425A-GGA derivative with P_GAL10_-*Sfp*-T_ADH1_; P_GAL1_-*EntD*-T_CYC1_ |
| pRS423-MetK/OMT | pRS423A-GGA derivative with P_GAL10_-*MetK*-T_ADH1_; P_GAL1_-*OMT*-T_CYC1_ |
| pRS423-MetK^I303V^/OMT | pRS423A-GGA derivative with P_GAL10_-*MetK^I303V^*-T_ADH1_; P_GAL1_-*OMT*-T_CYC1_ |
| pRS425-GapC/Pos5c | pRS425A-GGA derivative with P_GAL10_-*GapC*-T_ADH1_; P_GAL1_-*Pos5c*-T_CYC1_ |
| pRS425-CAR/PPTase | pRS425A-GGA derivative with P_GAL10_-*CAR*-T_ADH1_; P_GAL1_-*PPTase*-T_CYC1_ |
| pRS423-HpaB/HpaC | pRS423A-GGA derivative with P_GAL10_-*HpaB*-T_ADH1_; P_GAL1_-*HpaC*-T_CYC1_ |
| pRS425-BFD/HMO | pRS425A-GGA derivative with P_GAL10_-*BFD*-T_ADH1_; P_GAL1_-*HMO*-T_CYC1_ |
| pRS426-HmaS/OMT | pRS426A-GGA derivative with P_GAL10_-*HmaS*-T_ADH1_; P_GAL1_-*OMT*-T_CYC1_ |
| pRS426-UbiC/PobA | pRS426A-GGA derivative with P_GAL10_-*UbiC*-T_ADH1_; P_GAL1_-*PobA*-T_CYC1_ |
| pRS423-Xfpk/Pta | pRS423A-GGA derivative with P_GAL10_-*Xfpk*-T_ADH1_; P_GAL1_-*Pta*-T_CYC1_ |

**Table S4** Strains used in this study.

| **Name** | **Description** |
| --- | --- |
| JS-CR(2M) ^3^ | Strain BY4741 derivative with Δ*aro10*::P_GAL10_-*Aro4^fbr^*-T_CYC1_ and P_GAL1_-*Aro8*-T_CYC1_; Δ*trp1*:: P_GAL10_-*Aro7^fbr^*-T_CYC1_ and P_GAL1_-*Aro8*-T_CYC1_; P_CUP1_-*Gal4*; P_CTR1_-*Gal80* |
| JS-MARE1 | Strain JS-CR(2M) derivative with ∆*adh6*, ∆*adh7*, ∆*sfa1*, ∆*gre2* and ∆*hfd1* |
| JS-MARE2 | Strain JS-MARE1 derivative with ∆*gre3*, ∆*ydl124w*, ∆*gcy1*, and ∆*ypr1* |
| JS-MARE3 | Strain JS-MARE2 derivative with ∆*ari1*, ∆*ydr541c*, and ∆*aad3* |
| JS-CR(2M)-P1 | Strain JS-CR(2M) transformed with pRS423-AroZ/OMT and pRS425-CAR/PPTase |
| JS-MARE3-P2 | Strain JS-MARE3 transformed with pRS423-AroZ/OMT and pRS425-CAR/PPTase |
| JS-MARE3-P3 | Strain JS-MARE3 transformed with pRS423-HpaB/HpaC, pRS425-BFD/HMO, and pRS426-HmaS/OMT |
| JS-MARE3-P4 | Strain JS-MARE3 transformed with pRS425-CAR/PPTase and pRS426-UbiC/PobA |
| JS-VA1a | Strain JS-MARE3 derivative with Δ*rox1*::P_GAL10_-*AroZ*-T_ADH1_; P_GAL1_-*OMT*-T_CYC1_, Δ*bts1*::P_GAL10_-*CAR*-T_ADH1_; P_GAL1_-*PPTase*-T_CYC1_ |
| JS-VA1b | Strain JS-MARE3 derivative with Δ*rox1*::P_GAL10_-*AsbF*-T_ADH1_; P_GAL1_-*OMT*-T_CYC1_, Δ*bts1*::P_GAL10_-*CAR*-T_ADH1_; P_GAL1_-*PPTase*-T_CYC1_ |
| JS-VA2 | Strain JS-MARE3 derivative with Δ*rox1*::P_GAL10_-*AroZ*-T_ADH1_; P_GAL1_-*OMT*-T_CYC1_, Δ*bts1*::P_GAL10_-*CAR*-T_ADH1_; P_GAL1_-*PPTase*-T_CYC1_, Δ*ypl062w*::P_GAL10_-*Sfp*-T_ADH1_; P_GAL1_-*EntD*-T_CYC1_ |
| JS-VA3 | Strain JS-MARE3 derivative with Δ*rox1*::P_GAL10_-*AroZ*-T_ADH1_; P_GAL1_-*OMT*-T_CYC1_, Δ*bts1*::P_GAL10_-*CAR*-T_ADH1_; P_GAL1_-*PPTase*-T_CYC1_, Δ*ypl062w*::P_GAL10_-*Sfp*-T_ADH1_; P_GAL1_-*EntD*-T_CYC1_, Δ*yjl064w*::P_GAL10_-*GapC*-T_ADH1_; P_GAL1_-*Pos5c*-T_CYC1_ |
| JS-VA4 | Strain JS-MARE3 derivative with Δ*rox1*::P_GAL10_-*AroZ*-T_ADH1_; P_GAL1_-*OMT*-T_CYC1_, Δ*bts1*::P_GAL10_-*CAR*-T_ADH1_; P_GAL1_-*PPTase*-T_CYC1_, Δ*ypl062w*::P_GAL10_-*Sfp*-T_ADH1_; P_GAL1_-*EntD*-T_CYC1_, Δ*yjl064w*::P_GAL10_-*MetK*-T_ADH1_; P_GAL1_-*OMT*-T_CYC1_ |
| JS-VA5 | Strain JS-MARE3 derivative with Δ*rox1*::P_GAL10_-*AroZ*-T_ADH1_; P_GAL1_-*OMT*-T_CYC1_, Δ*bts1*::P_GAL10_-*CAR*-T_ADH1_; P_GAL1_-*PPTase*-T_CYC1_, Δ*ypl062w*::P_GAL10_-*Sfp*-T_ADH1_; P_GAL1_-*EntD*-T_CYC1_, Δ*yjl064w*::P_GAL10_-*MetK^I303V^*-T_ADH1_; P_GAL1_-*OMT*-T_CYC1_ |
| JS-VA6 | Strain JS-MARE3 derivative with Δ*rox1*::P_GAL10_-*AroZ*-T_ADH1_; P_GAL1_-*OMT*-T_CYC1_, Δ*bts1*::P_GAL10_-*CAR*-T_ADH1_; P_GAL1_-*PPTase*-T_CYC1_, Δ*ypl062w*::P_GAL10_-*Sfp*-T_ADH1_; P_GAL1_-*EntD*-T_CYC1_, Δ*yjl064w*::P_GAL10_-*MetK^I303V^*-T_ADH1_; P_GAL1_-*OMT*-T_CYC1_, Δ*pdc6*::P_GAL10_-*GapC* -T_ADH1_; P_GAL1_-*Pos5c*-T_CYC1_ |
| JS-VA7 | Strain JS-MARE3 derivative with Δ*rox1*::P_GAL10_-*AroZ*-T_ADH1_; P_GAL1_-*OMT*-T_CYC1_, Δ*bts1*::P_GAL10_-*CAR*-T_ADH1_; P_GAL1_-*PPTase*-T_CYC1_, Δ*ypl062w*::P_GAL10_-*Sfp*-T_ADH1_; P_GAL1_-*EntD*-T_CYC1_, Δ*yjl064w*::P_GAL10_-*MetK^I303V^*-T_ADH1_; P_GAL1_-*OMT*-T_CYC1_, Δ*pdc6*::P_GAL10_-*GapC* -T_ADH1_; P_GAL1_-*Pos5c*-T_CYC1_, Δ*pha2*::P_GAL10_-*HmaS*-T_ADH1_; P_GAL1_-*OMT*-T_CYC1_, Δ*are1*::P_GAL10_-*BFD*-T_ADH1_; P_GAL1_-*HMO*-T_CYC1_, Δ*gdh1*::P_GAL10_-*HpaB*-T_ADH1_; P_GAL1_-*HpaC*-T_CYC1_ |
| JS-VA8 | Strain JS-MARE3 derivative with Δ*rox1*::P_GAL10_-*AroZ*-T_ADH1_; P_GAL1_-*OMT*-T_CYC1_, Δ*bts1*::P_GAL10_-*CAR*-T_ADH1_; P_GAL1_-*PPTase*-T_CYC1_, Δ*ypl062w*::P_GAL10_-*Sfp*-T_ADH1_; P_GAL1_-*EntD*-T_CYC1_, Δ*yjl064w*::P_GAL10_-*MetK^I303V^*-T_ADH1_; P_GAL1_-*OMT*-T_CYC1_, Δ*pdc6*::P_GAL10_-*GapC* -T_ADH1_; P_GAL1_-*Pos5c*-T_CYC1_, Δ*gdh1*::P_GAL10_-*UbiC* -T_ADH1_; P_GAL1_-*PobA*-T_CYC1_ |

| JS-VA9 | Strain JS-MARE3 derivative with Δ*rox1*::P_GAL10_-*AroZ*-T_ADH1_; P_GAL1_-*OMT*-T_CYC1_, Δ*bts1*::P_GAL10_-*CAR*-T_ADH1_; P_GAL1_-*PPTase*-T_CYC1_, Δ*ypl062w*::P_GAL10_-*Sfp*-T_ADH1_; P_GAL1_-*EntD*-T_CYC1_, Δ*yjl064w*::P_GAL10_-*MetK^I303V^*-T_ADH1_; P_GAL1_-*OMT*-T_CYC1_, Δ*pdc6*::P_GAL10_-*GapC* -T_ADH1_; P_GAL1_-*Pos5c*-T_CYC1_, Δ*gdh1*::P_GAL10_-*UbiC* -T_ADH1_; P_GAL1_-*PobA*-T_CYC1_, Δ*gpp1*::P_GAL10_-*Xfpk*-T_ADH1_; P_GAL1_-*Pta*-T_CYC1_ |
| --- | --- |
| JS-VA10 | Strain JS-MARE3 derivative with Δ*rox1*::P_GAL10_-*AroZ*-T_ADH1_; P_GAL1_-*OMT*-T_CYC1_, Δ*bts1*::P_GAL10_-*CAR*-T_ADH1_; P_GAL1_-*PPTase*-T_CYC1_, Δ*ypl062w*::P_GAL10_-*Sfp*-T_ADH1_; P_GAL1_-*EntD*-T_CYC1_, Δ*yjl064w*::P_GAL10_-*MetK^I303V^*-T_ADH1_; P_GAL1_-*OMT*-T_CYC1_, Δ*pdc6*::P_GAL10_-*GapC* -T_ADH1_; P_GAL1_-*Pos5c*-T_CYC1_, Δ*gdh1*::P_GAL10_-*UbiC*-T_ADH1_; P_GAL1_-*PobA*-T_CYC1_, Δ*gpp1*::P_GAL10_-*Xfpk*-T_ADH1_; P_GAL1_-*Pta*-T_CYC1_, ΔP*_GAL80_ -GAL80*::P*_CTR1_*-*K15*∼*GAL80* |

**References**

1. DiCarlo, J.E. et al. Genome engineering in *Saccharomyces cerevisiae* using CRISPR-Cas systems. *Nucleic Acids Res* **41**, 4336-4343 (2013).

2. Mo, Q., Song, W., Xue, Z. & Yuan, J. Multi-level engineering of *Saccharomyces cerevisiae* for the synthesis and accumulation of retinal. *Green Chem* **24**, 8259-8263 (2022).

3. Fan, C., Zhang, D., Mo, Q. & Yuan, J. Engineering *Saccharomyces cerevisiae*-based biosensors for copper detection. *Microb Biotechnol* **15**, 2854-2860 (2022).
